# Mechanochemistry of Spiropyran under Internal Stresses of a Glassy Polymer

**DOI:** 10.1021/jacs.2c11280

**Published:** 2022-12-12

**Authors:** Richard Janissen, Georgy A. Filonenko

**Affiliations:** † Single-Molecule Biophysics, Department of Bionanoscience, Delft University of Technology, van der Maasweg 9, Delft 2629HZ, The Netherlands; ‡ Department of Materials Science and Engineering, Delft University of Technology, Mekelweg 2, Delft 2628 CD, The Netherlands

## Abstract

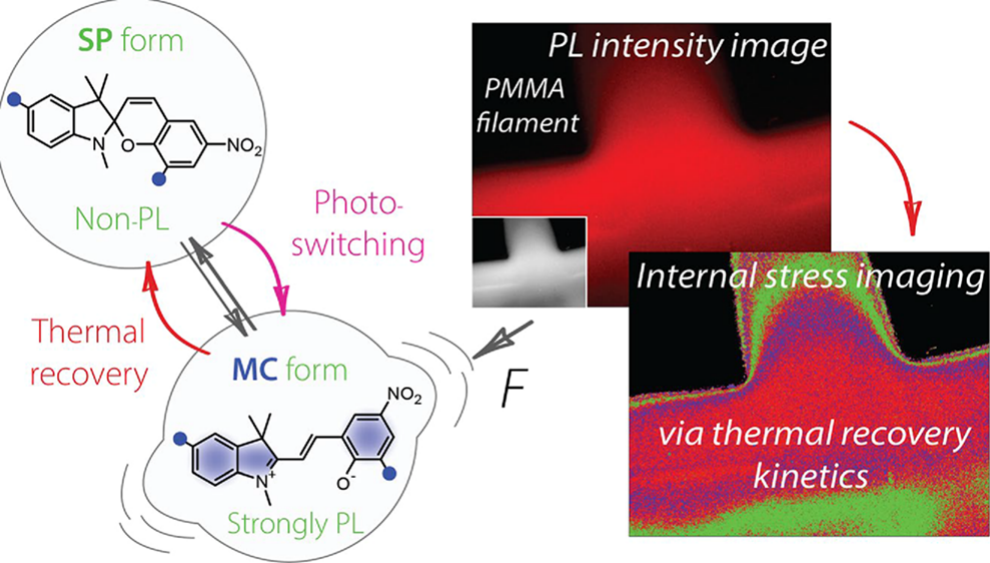

Mechanophores are powerful molecular tools used to track bond rupture and characterize mechanical damage in polymers. The majority of mechanophores are known to respond to external stresses, and we report in this study the first precedent of a mechanochemical response to internal, residual stresses that accumulate during polymer vitrification. While internal stress is intrinsic to polymers that can form solids, we demonstrate that it can dramatically affect the mechanochemistry of spiropyran probes and alter their intramolecular isomerization barriers by up to 70 kJ mol^–1^. This new behavior of spiropyrans (SPs) enables their application for analysis of internal stresses distribution and their mechanochemical characterization on the molecular level. Spectroscopy and imaging based on SP mechanochemistry showed high topological sensitivity and allowed us to discern different levels of internal stress impacting various locations along the polymer chain. The nature of the developed technique allows for wide-field imaging of stress heterogeneities in polymer samples of irregular shapes and dimensions, making it feasible to directly observe molecular-level manifestations of mechanical stresses that accompany the formation of a vast number of solid polymers.

## Introduction

**Figure 1:**
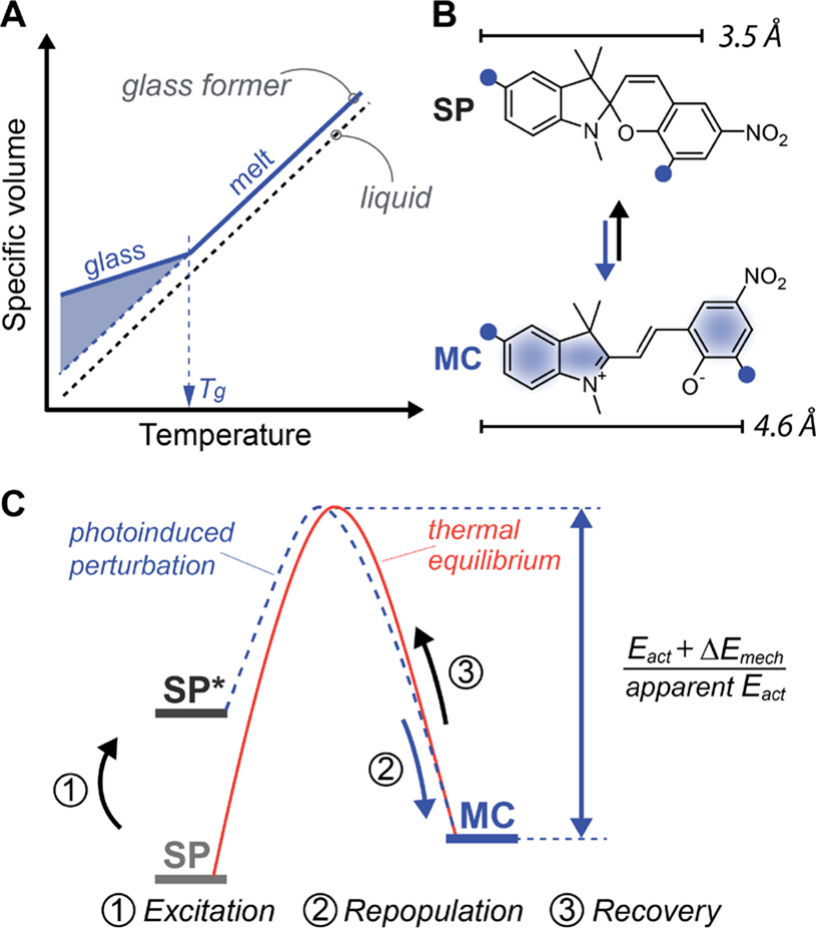
(A) Specific volume-temperature phase diagram of a polymer glass former showing the onset of vitrification at a glass transition temperature (*T*_g_) associated with the formation of internal stresses. (B) Spiropyran molecular probes and their isomerization kinetics and associated energy barriers (C) measured in this work by tracking SP/MC population and recovery.

Nearly all performance polymers experience mechanical stresses within their lifetime. Decades of research in polymer mechanochemistry^1,2^ have been targeted at studying manifestations of mechanical stress at the molecular level and revealed a plethora of stress-induced molecular phenomena. A large fraction of this progress can be attributed to the use of mechanophores—small force-sensitive molecules that are some of the most common molecular probes used for the investigation of stress-induced polymer transformations. A staple example of a mechanophore is the spiropyran (SP) probe that can undergo reversible ring opening, producing an intensely colored luminescent merocyanine (MC) isomer (Figure 1).^3^ Mechanically induced ring opening of SPs has been previously applied to study the deformation of various polymers, including PDMS,^4^ PMA^5^ and glassy PMMA,^6,7^ and polycarbonates,^8^ among other examples.^9^ Almost universally, SP-MC isomerization allows for tracking the extent of bond scission in the SP unit leading to the formation of MC upon application of external force or identical phenomena induced by physical swelling^10,11^ of polymer networks in the absence of external stresses. While the latter two are common types of extrinsic stresses known to bias mechanophore behavior,^12,13^ this work aims at investigating the stresses that are intrinsic and common for the majority of polymers in their solid state—internal or residual stresses.

Internal or residual stresses most commonly accumulate in polymers as they solidify from their melts or, in some specific cases, crystallize from it.^14,15^ As the formation of polymer solids is ubiquitous, so is the phenomenon of internal stress—it contributes to the behavior of polymer parts produced by common techniques like extrusion, 3D printing, and injection molding. The distribution of internal stresses and their magnitude within the polymer can be investigated both theoretically and experimentally. Experimental characterizations usually make use of optical phenomena, i.e., birefringence^16^ and photoelasticity,^17−19^ which, however, are not able to reveal the molecular-level information on the magnitude of stresses and their local heterogeneities in a real polymer.

In this study, we found that the molecular-level behavior of SP mechanophores is linked to internal stresses in glassy polymers. Having observed that internal stresses can dramatically affect the isomerization kinetics of SPs, we analyze the activation barriers for this reversible isomerization and use them to estimate the magnitude and spatial distribution of internal stresses. We further demonstrate that our technique is topologically sensitive and that the placement of SP in PMMA can affect the magnitude of the probe the response to vitrification or even completely invert the photochemical equilibrium in the SP–MC pair. Apart from the developed spectroscopy method, the discovered mechanochemistry can be used to perform wide-field imaging to reveal micro- and macroscopic stress heterogeneities in a simple and practical setting.

## Results and Discussion

At the onset of this investigation, we sought out to answer the fundamental question of whether the kinetics of small-molecule transformations is affected by vitrification. SPs serve as excellent probes for this task due to the high thermal and chemical robustness and the ease of the spectroscopic analysis of their isomerization to MCs. We hypothesized that if mechanical forces or, more generally, stresses emerge as a result of the formation of the solid polymer, this could affect the SP–MC transformation kinetics to a detectable extent. Specifically, we expected to detect alterations in the activation barriers of the SP–MC transformation resulting from compressive or tensile forces that might create an additional kinetic bias for either isomerization direction. In part, our choice of using SP was supported by the work of Sottos and co-workers who reported that externally applied tensile stresses can perturb the SP isomerization kinetics in thermoplastic polymers.^20^ The authors have found that external stresses up to tens of MPa applied at a constant temperature inflict a change in isomerization rates that translated to a few kJ mol^–1^ difference in the activation energy *E*_act_. To render our investigation analytically robust, we studied the temperature dependence of the SP isomerization kinetics that would provide a direct measurement of the activation barrier of the SP–MC transformation.

**Figure 2:**
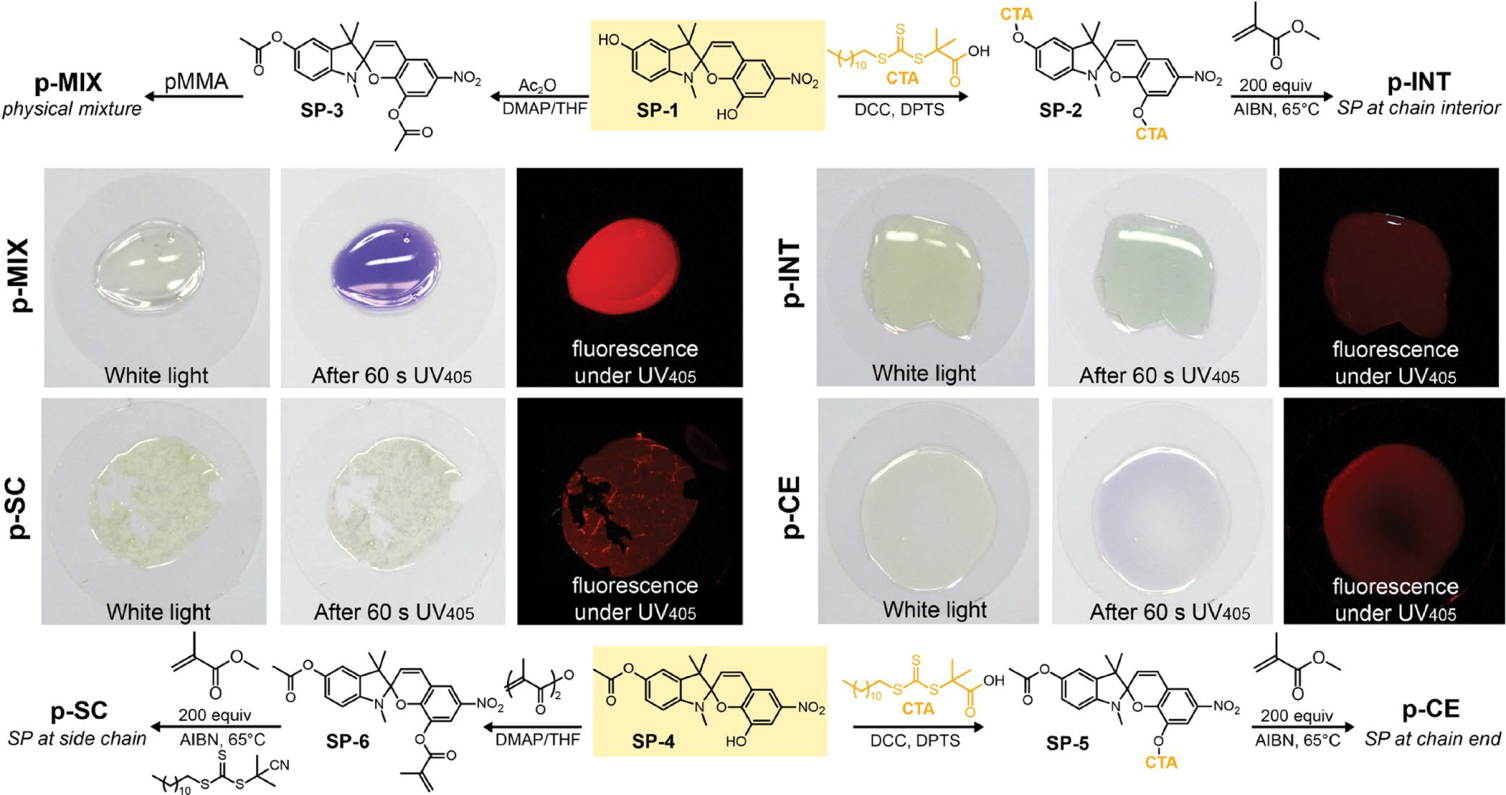
Synthesis of PMMA samples with different SP placement: physical mixture of PMMA and **SP-3** (**p-MIX**), chain interior (**p-INT**), random side chain (**p-SC**), and chain end (**p-CE**). Photographs of the dried samples stored in ambient white light (left), immediately after the exposure to UV for 60 s (middle), and under UV light (right, 405 nm, photographed through the long-pass filter). See Section 2 of the Supporting Information for synthesis details and abbreviations, and Figure S33 for color-progression images.

Four sets of samples were prepared for this study, all featuring the derivatives of the common nitro spiropyran **SP-1** (Figure 2; see the Supporting Information for synthesis details and compound descriptions) as a sensory molecule. The sample containing the SP unit in the chain interior (**p-INT**) was prepared via RAFT polymerization using difunctional **SP**-**2** as a chain transfer agent. We next synthesized PMMA samples with SP units located at the chain end (**p-CE**) or randomly introduced into the *sidechain* of PMMA (**p-SC**). These samples contained monofunctional SP derived from **SP-4** (Figure 2) that ensures a single point of connection to the polymer chain. As the last sample, we prepared a *physical mixture* (**p-MIX**) of the PMMA polymer and **SP-3** featuring no covalent connection between the polymer and the SP unit. All samples were drop-cast from chloroform solution, dried under vacuum at 65 °C overnight, and annealed at 125 °C for 10 min before analysis.

Examining the behavior of PMMA samples under white and UV light (405 nm), we noticed qualitative differences in their ability to sustain SP photoisomerization. While the physically mixed SP (**p-MIX**) readily converted to the MC form within 60 s of exposure to UV light, the **p-SC** and **p-INT** samples exhibited suppressed SP activation (Figure 2). Notably, the **p-CE** sample with the SP unit located at the chain end showed a significant degree of MC formation, despite the fact that the SP unit was covalently attached to the polymer chain. Having observed these differences, we sought out to investigate the SP–MC isomerization kinetics. In a purely thermal setting, SP–MC transformation kinetics in the solid polymers is challenging to study as the time necessary to thermally equilibrate the samples might bias the accuracy of the measurements. Instead, we utilized the approach similar to that used by the groups of Sottos^20^ and Craig,^21^ who tracked the thermal recovery of SPs following photoinduced isomerization: once the SP-containing samples are thermally equilibrated, one can perturb the thermal equilibrium by the irradiation with UV light (375 nm), shifting it to the MC form. When the irradiation stops the SP equilibrium population would recover and the recovery kinetics can then be readily monitored using absorption spectroscopy.

**Figure 3:**
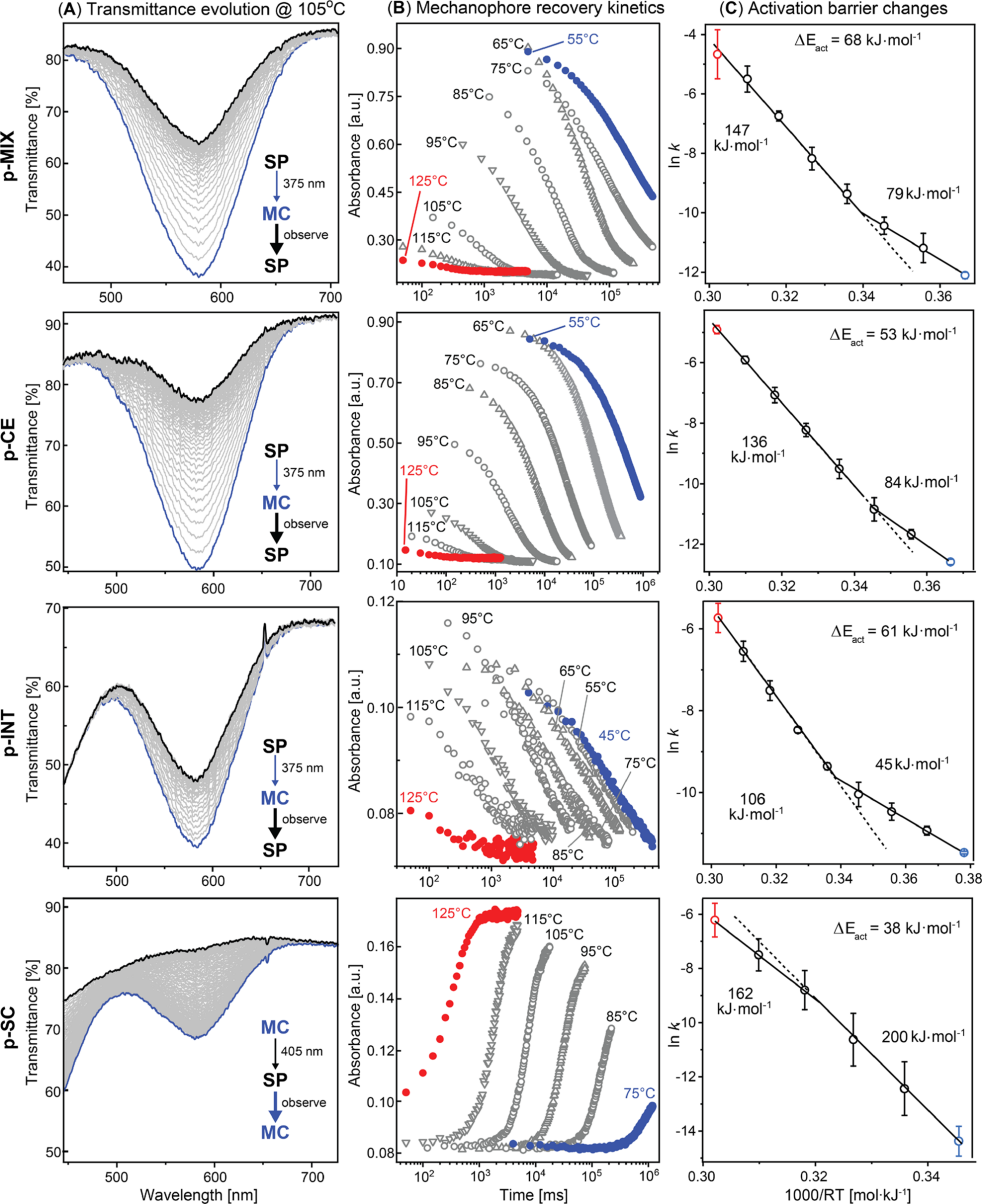
Principal data for MC–SP equilibria establishment kinetics for PMMA samples with SP added to different locations. (A) Examples of transmittance evolution profiles at 105 °C. (B) Kinetic traces for absorbance decay at 573 nm in a broad temperature–time range; note the log scale used for the time axis to depict a timespan of several orders of magnitude. (C) Arrhenius plots depicting activation barrier changes. See also Section 3 of the Supporting Information for details.

We found that vitrification had a strong impact on the SP–MC isomerization kinetics in all tested samples (Figure 3). For example, in **p-MIX** above the glass transition, a decrease of the rate constant in a temperature ranging from 125 to 85 °C in PMMA-SP mixtures was consistent with an activation energy *E*_act_ of 147 kJ mol^–1^ for MC ring closure (MC to SP, Figure 3). Upon vitrification, however, *E*_act_ decreases further to 79 kJ mol^–1^, indicating that the mechanical stress reduced the activation barrier of this reaction by a large value of 68 kJ mol^–1^. Given that the ring closure reaction monitored in this experiment was associated with molecular contraction, we concluded that the change in *E*_act_ was associated with *compressive stresses* developed during vitrification. In studies focused on tensile elongation, the changes in activation barriers are commonly analyzed using the Bell–Evans model, as used previously by the groups of Craig and Sottos.^20,21^ The value of ∼260 pN reported by Craig and co-workers^21^ using single-molecule force spectroscopy to probe the MC ring-opening reaction would correspond to an activation barrier change of 72 kJ mol^–1^—a value close to the *E*_act_ observed in this work.

The literature on residual stress distribution in polycarbonates and PMMA reports the presence of compressive stresses up to 20 MPa at the sample surface.^15^ Similar values of 30–40 MPa were also reported recently for polystyrene spin cast films.^22^ The direct calculation of mechanical stress from the observed mechanophore equilibration kinetics is challenging since it requires proper model assumptions and accurate analysis of molecular-level forces. Nonetheless, one can make use of the measured kinetics to estimate the magnitude of acting mechanical stress. Assuming that molecular-level forces, similar to those observed by Craig,^21^ act on a mechanophore molecule encased in the spherical free volume element of PMMA, one can use the estimation of the free volume element radius in PMMA of ca. 5 Å from Fayer and co-workers.^23,24^ Using this value, we estimated a stress of ca. 20.7 MPa that is placed on the surface of the 5 Å-radius sphere by a force of 260 pN, which is close to the literature values reported previously.^21^

We next observed that the covalent attachment of the SP unit to the PMMA chain significantly affected the mechanophore vitrification response. For example, the **p-CE** sample that was labeled at the chain end featured lower *E*_act_ of ∼136 kJ mol^–1^ in the melt state, indicating that even in monofunctional SPs the isomerization was affected by the presence of the polymer chain in the melt. Compared to **p-MIX**, this sample showed a somewhat lower magnitude of response to vitrification in our measurements with a Δ*E*_act_ of 53 kJ mol^–1^.

These findings are consistent with our recent report suggesting that polymer end groups might be confined to the free volume voids at the polymer chain end vicinity, partially separating the confined molecule from the host-induced behavior.^25^ Several reports from the group of Fayer estimate the average free volume element size radius in PMMA with ca. 5 Å, which is close to the MC activation length of 4.5 Å and indicates the possibility of MC confinement in chain-end labeled samples.^23,24^

A more profound example of the topology being able to affect the mechanophore behavior was observed when placing the mechanophore in the polymer backbone as in the **p-INT** samples. The activation barrier of 106 kJ mol^–1^ found for the MC ring closure in the melt state was significantly lower than that of mono- and non-functionalized SPs (**p-MIX** and **p-CE**), suggesting an additive effect of covalent incorporation of SPs in linear polymers. Vitrification, however, further decreased *E*_act_ to 45 kJ mol^–1^, indicating that the chain interior in PMMA is subject to much larger forces in absolute terms in both melt and glassy states.

Another topologically distinct location in PMMA is the side chain of the polymer. With the indication that thermal, mechanical, and rheological behavior is affected by the presence and properties of side chains,^26−28^ we expected the side chain of PMMA to be affected differently by vitrification compared to other polymer compartments. Indeed, the **p-SC** sample containing SP in the side chain, a location with high steric congestion, showed a strikingly different photochemical behavior compared to the other PMMA samples: the introduction of SP to the polymer side chain led to the partial reversal of the photochemical reactivity. Namely, the photoexcitation of **p-SC** samples with UV light (405 nm) promoted a ring closure reaction instead of a ring-opening reaction, i.e., the formation of the SP form instead of the MC form.

This behavior allowed us to monitor the reverse ring-opening reaction (SP to MC) associated with a mechanophore expansion that in the presence of compressive stresses should result in an increase of the apparent *E*_act_ as opposed to the previous samples where *E*_act_ was decreased. Indeed, the activation barrier of 162 kJ mol^–1^ observed in the melt state increased by 38 kJ mol^–1^ as the sample vitrifies to ca. 200 kJ mol^–1^. This result further confirms the compressive nature of internal stress, i.e., that vitrification inhibits reactions associated with extension and promotes reactions associated with contraction. This circumstance, on the other hand, suggests that mechanophores at the polymer side chain are subject to the lowest magnitude of internal stress. Together with results on SP–MC equilibration kinetics for other PMMA samples, the results for **p-SC** further suggest that molecular-level stress distribution is *topology-sensitive* as was our analysis technique.

**Figure 4:**
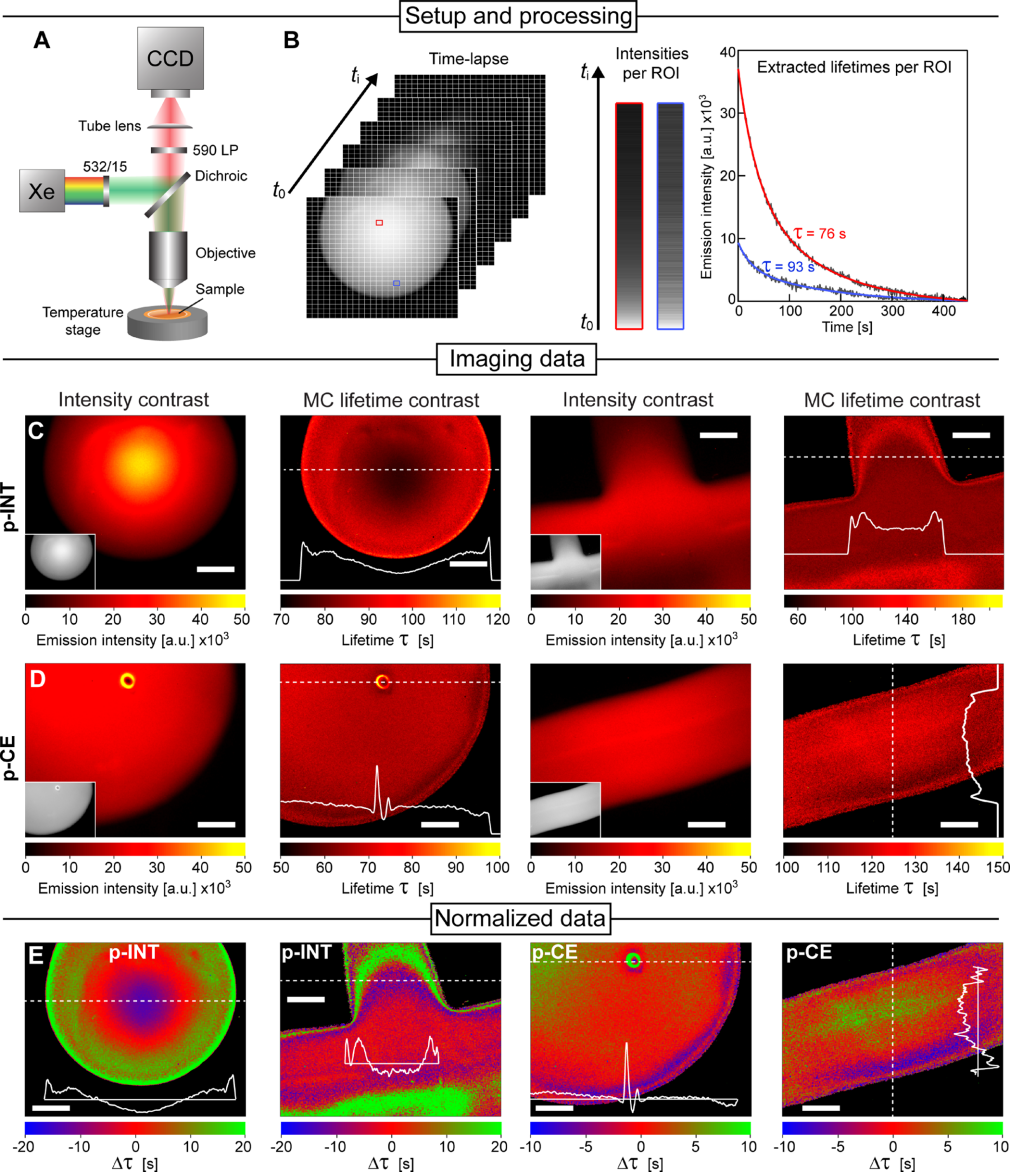
Thermal lifetime imaging of stress heterogeneities in **p-INT** and **p-CE** PMMA-SP samples. (A) Imaging setup schematics with 532/15 nm light irradiation of the PMMA samples and a 590 nm long-pass filter for PL measurement. (B) Image processing steps: extracting the intensity of each pixel from the time-lapse imaging data and performing mono-exponential fitting of the PL decay over time. Examples given for two regions (red and blue ROIs; 1 px per ROI). (C, D) Images of initial PL intensities (left) and heat maps of MC lifetime decays (right) for (C) **p-INT** and (D) **p-CE** samples with different shapes. (E) Heat maps of MC decay lifetimes as in (C) and (D), but normalized to the average integrated lifetime. All line profiles (white) correspond to MC lifetime decay data across the dotted white lines in the heat maps. Scale bars denote 200 μm.

Since the monitoring of the mechanophore recovery kinetics requires simple spectroscopic measurements, it can easily be expanded beyond spectroscopy and implemented in a wide-field imaging setting (Figure 4A). For example, the recovery of the photoactivated SP form can be monitored via the decay of the photoluminescence (PL) intensity produced by the MC form. In this work, however, we complemented conventional PL imaging with local kinetic measurements to extract thermal relaxation lifetimes. Unlike intensity data, this lifetime parameter is independent from the mechanophore concentration while reflecting the local stress state. Namely, high compressive stress would promote MC-to-SP ring closure, resulting in short lifetimes, while tension, expected to manifest on the edges of solidifying samples, would increase the observed MC thermal lifetime.

To demonstrate the feasibility of the technique, we performed the imaging using an epifluorescence microscope with green light excitation (λ = 532/15). Imaging at 65 °C—a temperature at which MC-recovery is rapid (see kinetic traces in Figure 3)—allowed the time-lapse measurement of the PL decay to be performed within 5 min. To characterize the MC decay kinetics based on the PL intensity data, we performed the following image analysis: the PL intensity was extracted for each pixel of the image over time (Figure 4B) and fitted to an exponential decay function, gaining the decay lifetimes (τ) of the photoluminescent MC form per pixel (Figure 4B). The lifetime heat maps were then constructed from the extracted lifetimes for each pixel (Figure 4C,D; right panels), which correspond to the MC–SP ring closure reaction lifetime, reciprocal to the reaction rate constant. The normalization of the heat maps to the average lifetime shown in Figure 4E further highlights areas of high (low lifetimes—fast MC decay) and low (high lifetimes—slow MC decay) compressive stresses in the different samples.

Similar to conventional lifetime imaging techniques, i.e., fluorescence-lifetime imaging microscopy, our data represent PL relaxation lifetimes rather than MC concentrations, rendering this imaging approach insensitive to irregularities in sample thickness or other imperfections. This property is evident when comparing the original intensity-based images with lifetime heat maps shown in Figure 4. We additionally studied **p-INT** and **p-CE** samples drop casts with hemispherical, round, cross, and filament shapes and observed stress heterogeneities in all of them. In line with the residual stress distribution observed in PMMA probe lifetime imaging indicates the accumulation of compressive stress immediately beneath the edge of all samples, which is expected for melt-quenched samples regardless of their shape.^15^ While our spectroscopic analysis shown in Figure 3 suggests that residual stresses are on average compressive in all samples, we also noticed a high level of heterogeneity in the samples. The imaging data revealed large deviations from the average MC decay lifetime within all samples: a two- to three-fold change in MC lifetime was observed within individual samples, indicating that potentially both tension and compression can manifest within glassy PMMA in line with previous reports.^15^ Clearly, the future development of this technique would require a more accurate analysis of mechanochemical reactions to obtain quantitative stress estimates. We believe that once the accurate analysis of molecular forces acting on the SP–MC pair in the glassy polymer could be performed, one would be able to perform such mapping with high resolution and chemical specificity.

## Conclusions

Our results demonstrate that the mechanochemistry and photochemical behavior of SPs enable the capture and evaluation of the development of internal stresses intrinsic to the majority of solid polymers. Our approach, based on characterizing the mechanophore thermal isomerization kinetics, provides direct evidence that internal stresses in PMMA can lead to the occurrence of mechanical stress that can dramatically affect the kinetic bias for mechanophore isomerization by up to 70 kJ mol^–1^—nearly half of the overall reaction barrier. Importantly, these stresses were shown to be topology dependent as the chain interior location within PMMA was affected most in both melt and glassy states. The effects of the local environment were found to be sufficiently strong to also affect the equilibrium behind mechanophore speciation: when the mechanophore was placed in an environment with higher steric congestion, the photochemical SP ring opening was completely suppressed in favor of MC ring closure. Finally, our findings and methodology enable highly informative and operationally simple measurements. With the combined use of spectroscopy and fluorescence imaging techniques, it is possible to survey polymeric solids for internal stresses, map their heterogeneity, localization, and analyze their magnitude. These first of a kind molecular-level observations of internal stresses in the vicinity of molecular probes confirm that internal stresses engage molecular probes and strongly affect their chemistry on the molecular length scales even in the absence of external forces.

## Supporting Information Available

Video recordings of photoswitching for p-CE (MP4)
Video recordings of photoswitching for p-INT (MP4)
Video recordings of photoswitching for p-MIX (MP4)
Video recordings of photoswitching for p-SC (MP4)
Synthesis procedures, characterization data for compounds and polymer samples, and methodology description for spectroscopy and imaging analysis (PDF)
