# Supplementary material for: Mechanochemistry of Spiropyran under Internal Stresses of a Glassy Polymer
Source: J Am Chem Soc. 2022 Dec 12;144(50):23198–204. doi: 10.1021/jacs.2c11280 (PMC9782785; doi:10.1021/jacs.2c11280)
Supplement: Supplementary file 5 — ja2c11280_si_005.pdf [file ja2c11280_si_005.pdf]

Supporting Information

Mechanochemistry of Spiropyran Under Internal Stresses of a Glassy Polymer

Richard Janissen[a] and Georgy A. Filonenko[b,]*

[a] Single-Molecule Biophysics, Department of Bionanoscience, Delft University of Technology, van der Maasweg 9, 2629HZ Delft, The Netherlands

[b] Department of Materials Science and Engineering, Delft University of Technology, Mekelweg 2, 2628 CD Delft, The Netherlands

* Corresponding author: Georgy A. Filonenko (g.a.filonenko@tudelft.nl)

KEYWORDS: mechanophores, residual stress, polymers, polymeric glass, stress imaging.

Table of Contents

1. General 2
2. Synthesis and characterization 3
3. Spectroscopy and imaging 43

Dataset for this publication is available from 4TU.Research data under DOI: 10.4121/20363643

1. General

Unless stated otherwise, all manipulations were performed in an Argon-filled glovebox (INERT, USA) or using the Schlenk technique. Anhydrous solvents were dispensed from a solvent purification system manufactured by INERT (USA), degassed prior use, and stored over 4 Å molecular sieves. Anhydrous deuterated solvents were purchased from Eurisotop, degassed, and stored over 4 Å molecular sieves. Methyl methacrylate was passed through an alumina plug, degassed, and used immediately. AIBN initiator was recrystallized from warm diethyl ether prior to use. Unless noted otherwise, all other chemicals were purchased from major commercial suppliers (TCI Europe and Sigma-Aldrich) and used without further purification.

Instrumentation

NMR spectra were measured with a Agilent 400-MR DD2 spectrometer. **LCMS measurements** were performed using a LTQ XL spectrometer equipped with a Shimadzu HPLC setup and operating at a 0.2 mL/min flow rate with a water/MeCN mobile phase and a Discovery C18 column. **Gel permeation chromatography** (GPC) measurements were performed using a Shimadzu HPLC setup equipped with a Agilent PLGel column operating at 40°C with a DMF/LiBr eluent at a 1 mL/min flow rate. Quantification was performed using a refractive index detector; the instrument was calibrated using ReadyCal PMMA standards. Glass transition temperatures were determined using DMA analysis via oscillatory shear geometry (1 Hz, 0.05% strain) in a temperature sweep mode (2.5°C/min, 65 to 130°C range), performed with TA HR-3 Rheometer with 8 mm plate fixture. Samples were conditioned at 135°C before the measurement for at least 10 minutes and the T_g value was estimated as a peak of the loss modulus.

Photophysical characterization

The **absorbance measurements** were performed using an Ocean Optics HDX spectrometer integrated in a free space fiber optics assembly with white light supplied by DH-MINI source (Ocean Optics, Deuterium/Halogen, fed through a 200 µm fiber with an ND0.5 filter).

Temperature control was performed with a modified Linkam THMS 600 sample stage. UV-induced equilibria redistributions were initiated using Thorlabs LEDs (375 and 405 nm), mounted within a distance of 20-25 mm of the sample and operating at full power.

Video files showing photoswitching were captured using iPhoneSE (A1723, Apple) cell phone camera for polymer samples incubated at ca. 110°C.

2. Synthesis and characterization

Chain interior PMMA (p-INT) and difunctional CTA SP-2

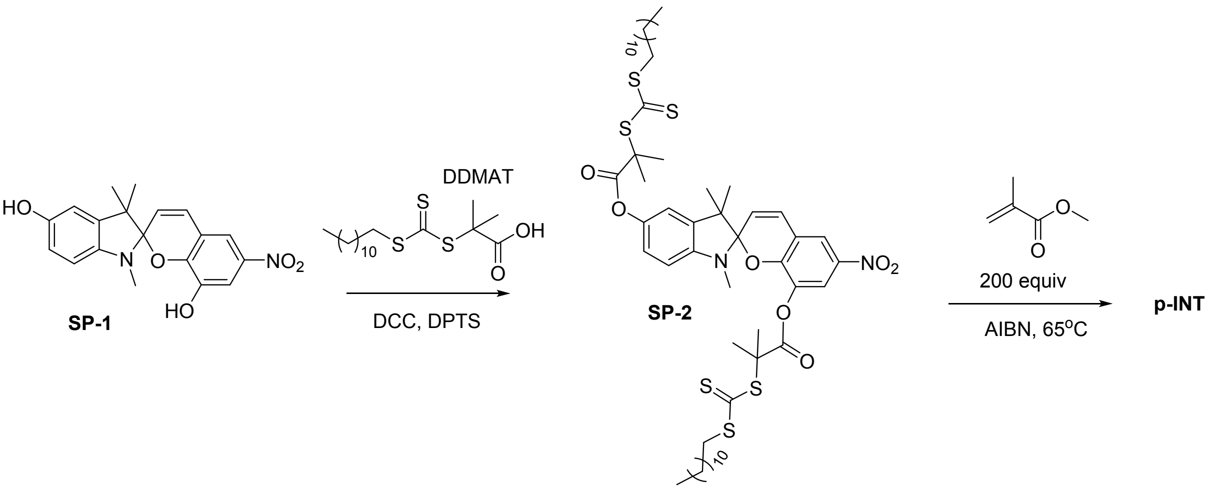

SP-2:

Spiropyran **SP-1** (354 mg, 1 mmol, 1 eq.), prepared according to literature procedure[1], was loaded as solid in a 20 mL vial in a glovebox together with 728 mg of solid DDMAT (2 mmol, 2 eq.), also prepared according to published procedure[2], and 558 mg (2 mmol, 2 eq.) of DPTS catalyst (this is a 1:1 salt of PTSA and DMAP, prepared according to Moore and Stupp[3]). 15 mL of DCM was added to these solids and the suspension was further stirred for 5 min to homogenize until a nearly clear dark purple solution was formed. Separately, 515 mg DCC (2.5 mmol, 2.5 eq.) was weighed out and added as a solution in DCM dropwise over 2-3 min to the main solution.

After 8 h, LC-MS indicated consumption of the starting material and the reaction mixture was taken out of the glovebox, filtered to remove the DCU precipitate (PP frit), and worked up as following: DCM filtrates were extracted with water (2x 5 mL), 0.2 M HCl (2x 5mL), saturated aq. $NaHCO_3$ (1x 10 mL). The organic extracts were then dried over sodium sulfate and passed through an alumina plug to yield 920 mg (88%) of a light purple thick oil that slowly turned yellow. The crude product contained traces of DCU and DDMAT-DCC N-acyl urea adducts and was further purified by column chromatography on a short silica plug in DCM (R_f 0.8, light yellow band, noted to stain silica purple). Purification of 120 mg of the crude oil yielded 90 mg of the pure **SP-2**.

NMR: ^{1}H NMR (400 MHz, $CDCl_3$) δ 7.94 (d, J = 2.7 Hz, 1H), 7.72 (d, J = 2.6 Hz, 1H), 6.98 (d, J = 10.4 Hz, 1H), 6.83 (dd, J = 8.3, 2.3 Hz, 1H), 6.76 (d, J = 2.3 Hz, 1H), 6.45 (d, J = 8.3 Hz, 1H), 5.90 (d, J = 10.4 Hz, 1H), 3.32 (td, J = 7.5, 3.0 Hz, 4H), 2.66 (s, 3H), 1.83 (overlap of 2 s, 3H each), 1.69 (m, 4H), 1.40 (m, 4H), 1.38 – 1.14 (m, overlap of 8x CH_2 DDMAT, 2x CH_3 DDMAT, 42H), 0.87 (t, J = 6.7 Hz, 6H).

^{13}C NMR (100 MHz, $CDCl_3$) δ 221.80, 221.55, 171.95, 170.33, 151.39, 145.41, 144.96, 140.27, 137.60, 137.27, 128.73, 120.86, 120.36, 120.31, 119.74, 119.42, 115.40, 107.69, 107.36, 55.97, 55.57, 51.85, 37.27, 37.22, 32.04, 29.77, 29.75, 29.74, 29.69, 29.67, 29.59, 29.57, 29.46, 29.27, 29.23, 29.10, 29.08, 28.88, 28.06, 28.03, 25.92, 25.53, 25.51, 24.59, 24.48, 22.81, 19.46, 14.25.

LC-MS: SP+H^+ 1047.00 (Calc. 1047.41). Does not elute on reverse phase; direct infusion used instead.

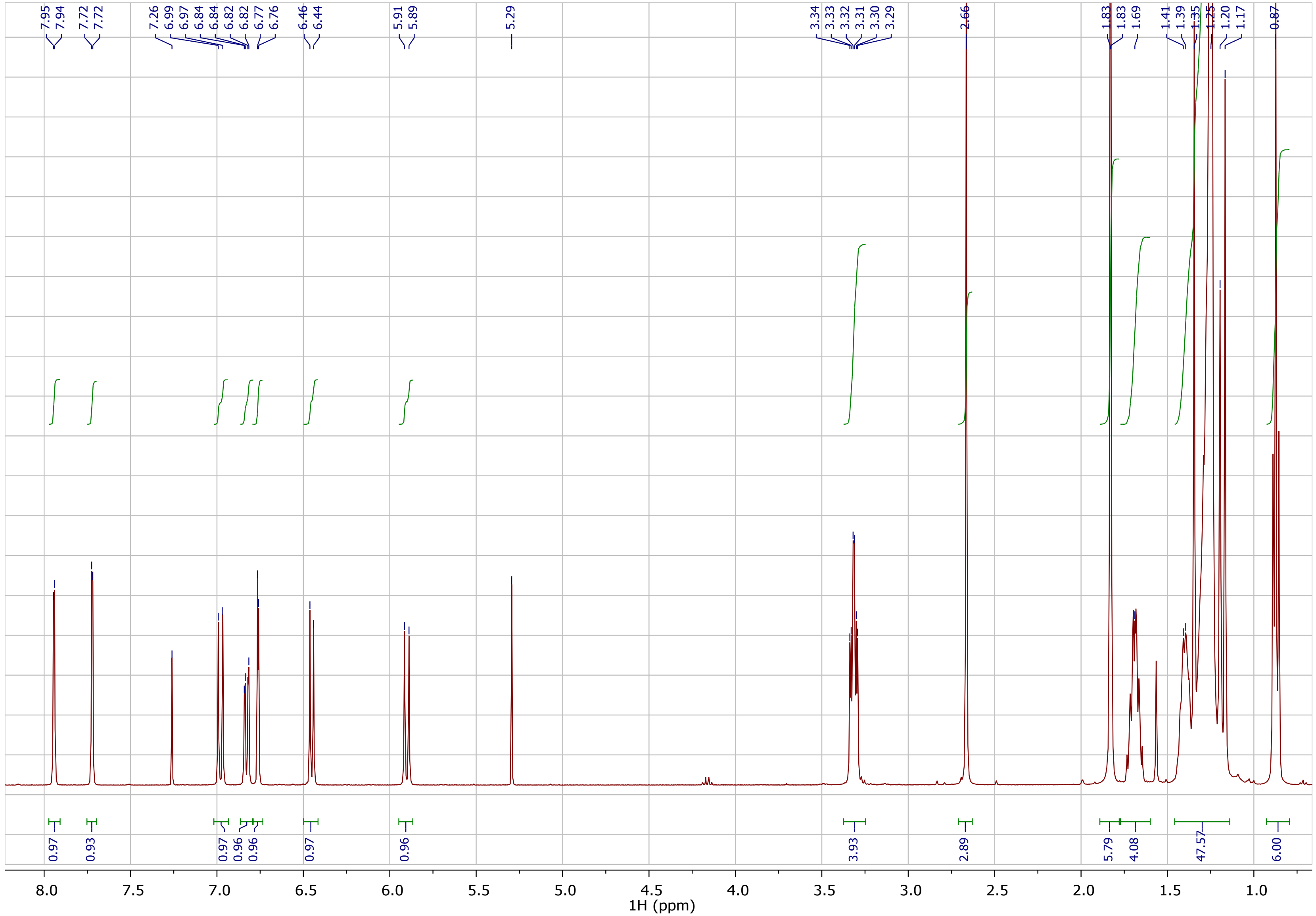

Figure S1. ^{1}H NMR spectrum of SP-2 ($CDCl_3$).

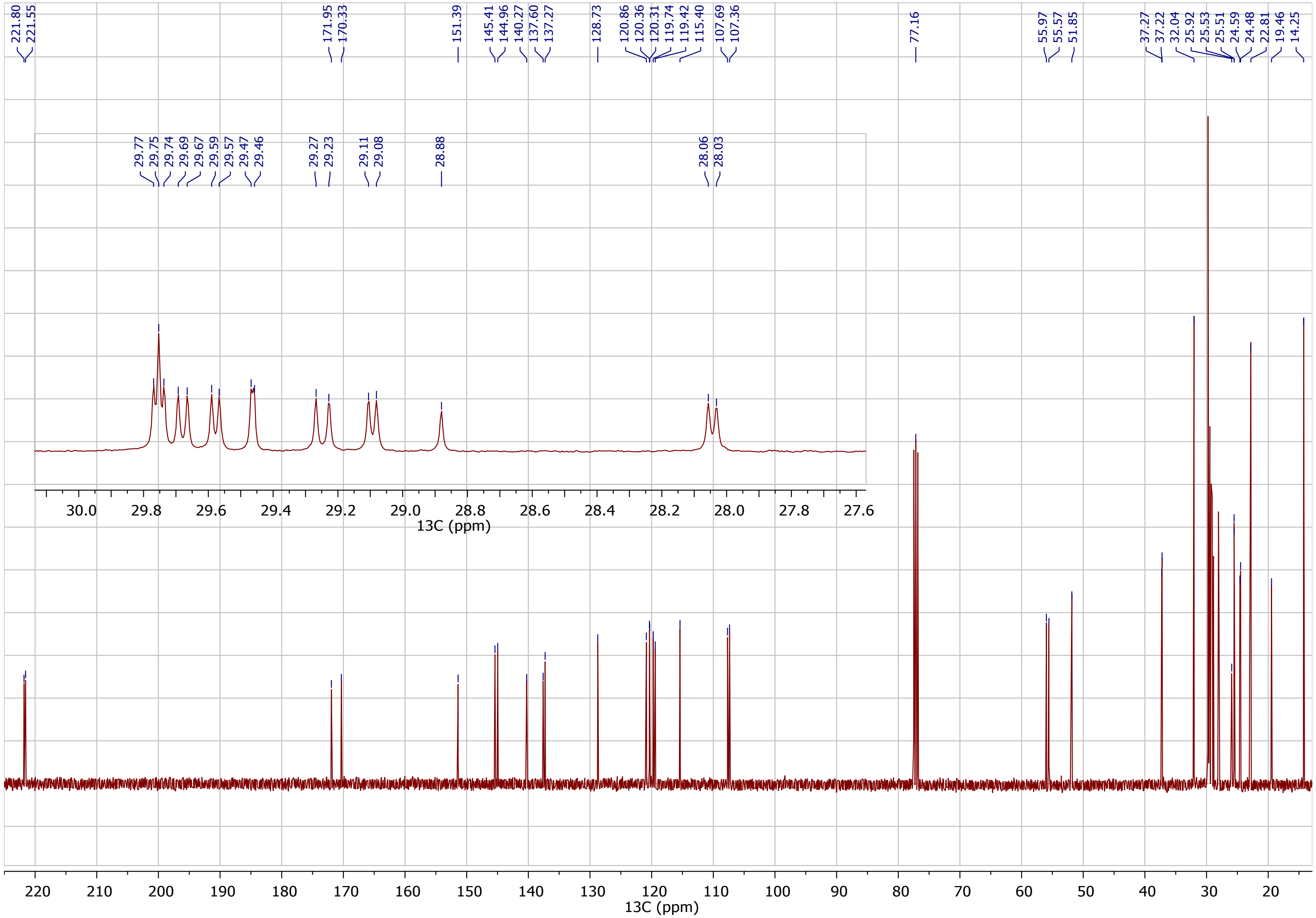

Figure S2. ^{13}C NMR spectrum of SP-2 ($CDCl_3$).

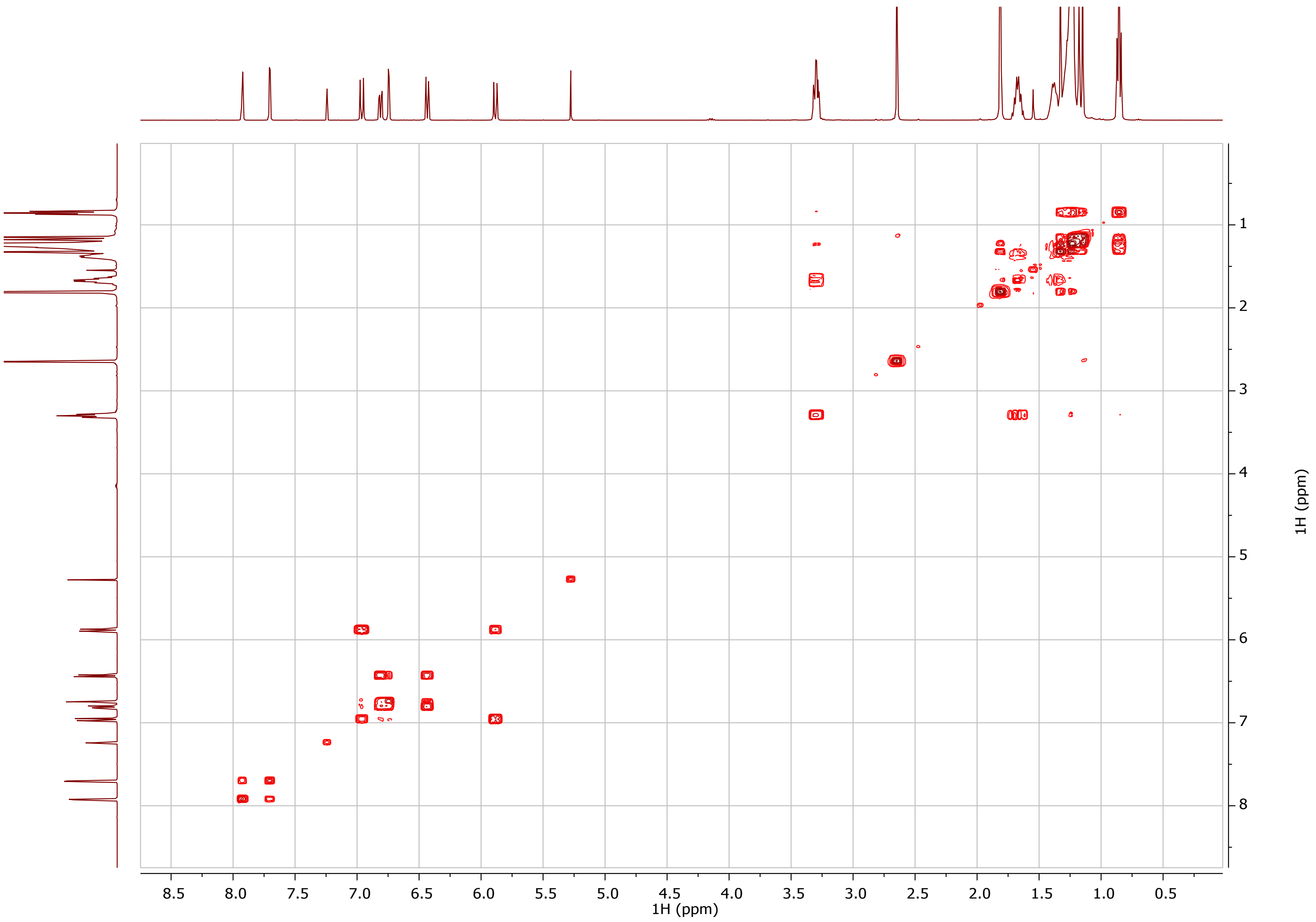

Figure S3. gCOSY spectrum of SP-2 ($CDCl_3$).

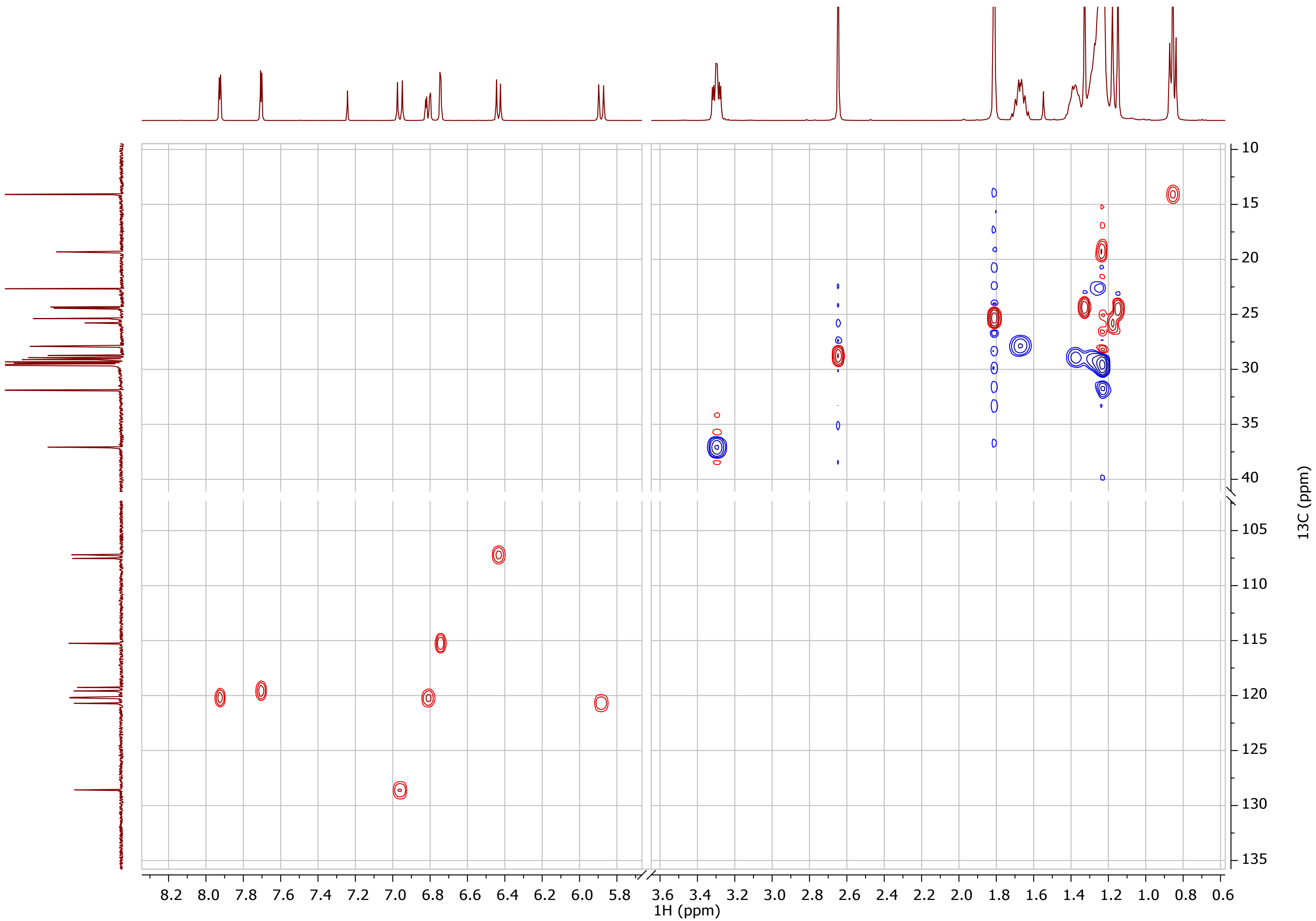

Figure S4. gHSQC spectrum of SP-2 ($CDCl_3$).

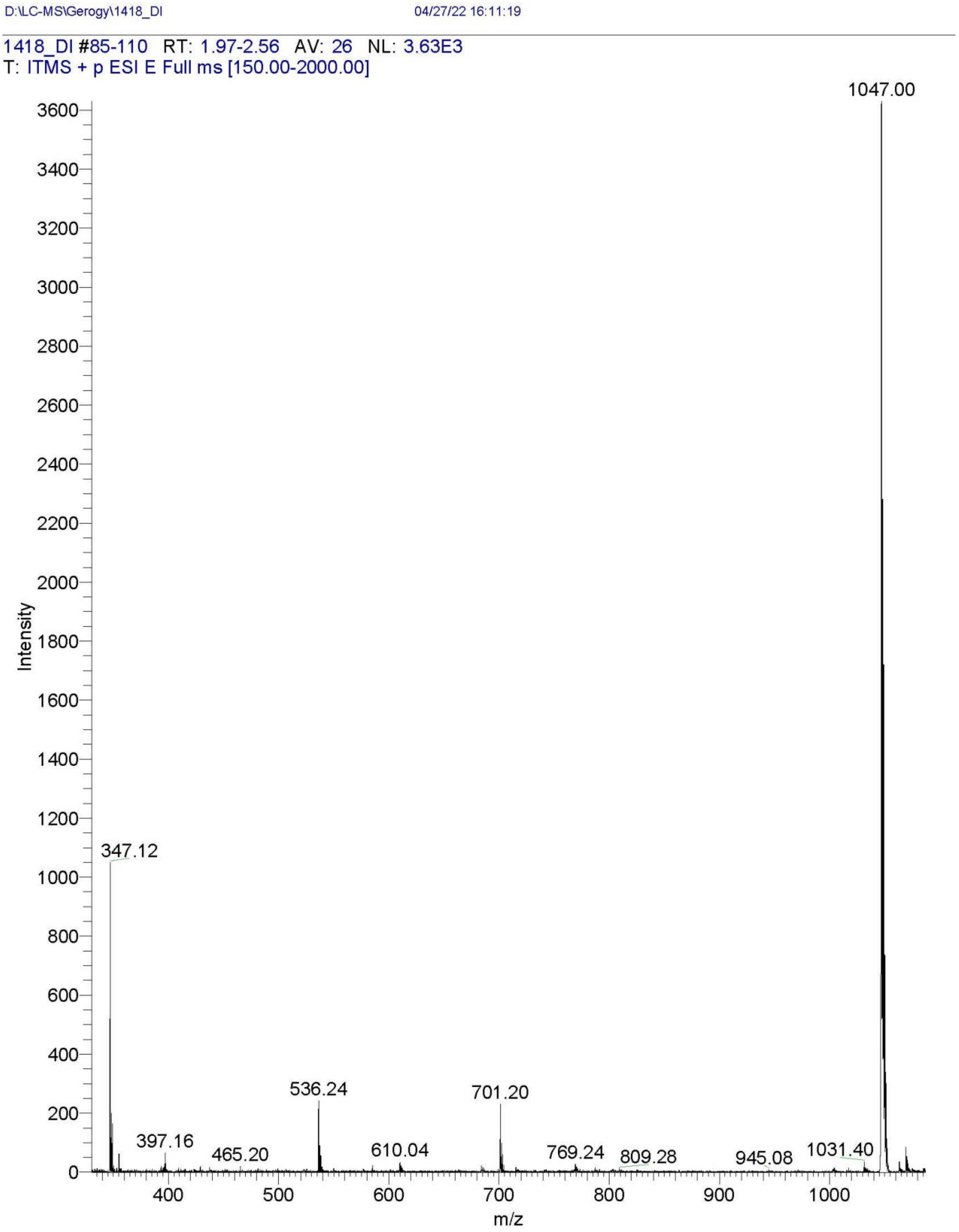

Figure S5. LC-MS data for SP-2.

Polymerization of SP-2: p-INT

Methyl methacrylate (0.5 mL, 4.694 mmol, ca. 300 eq) and 0.5 mL benzene-d_6 were added to the NMR tube containing 16.5 mg of SP-2 (0.01577 mmol, 1 eq.) and 0.5 mg of AIBN (0.003 mmol). The mixture was homogenized and set at 65°C overnight.

General workup:

The PMMA lump set in the tube was dissolved in 1 mL acetone and poured into stirring cold methanol (-20°C) to precipitate the polymer, which was separated by decantation, triturated in a new portion of methanol (room temperature), again decanted. The fine polymer powder formed was stirred in 10 mL methanol to complete the washing. Subsequently, methanol was decanted and the sample was washed with pentane and dried under vacuum (5 mbar, 50°C, 1 h).

A portion of 150 mg of dried p-INT polymer was analyzed using NMR spectroscopy and the solution in $CDCl_3$ was used directly for casting on a glass cover slips. Cover slips were covered with a glass lid to prevent rapid evaporation and after 4 hours the cover slips were dried in the vacuum oven at 65°C overnight (12-16 hours).

GPC: 32.13 kDa (PDI 1.51)

DMA: T_g = 100.05°C (1Hz)

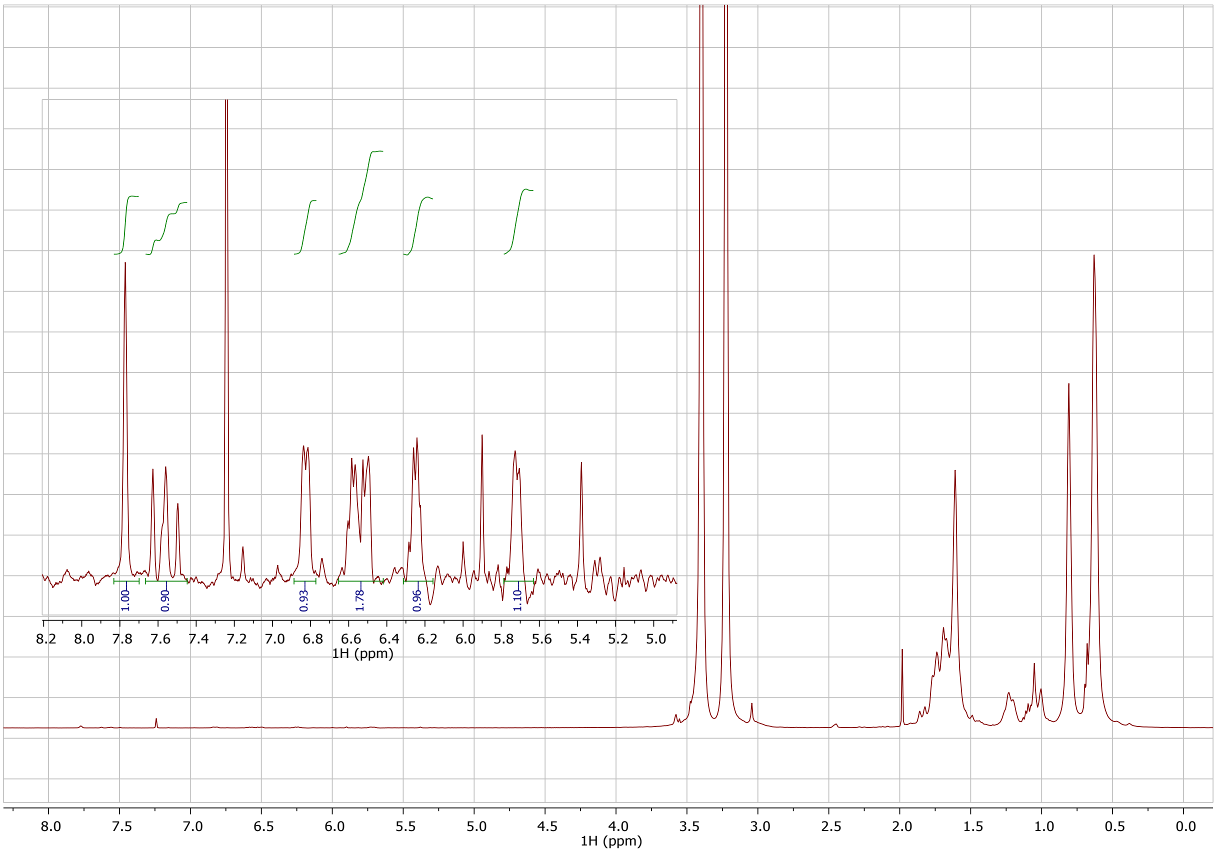

Figure S6. ^1H spectrum of p-INT ($CDCl_3$).

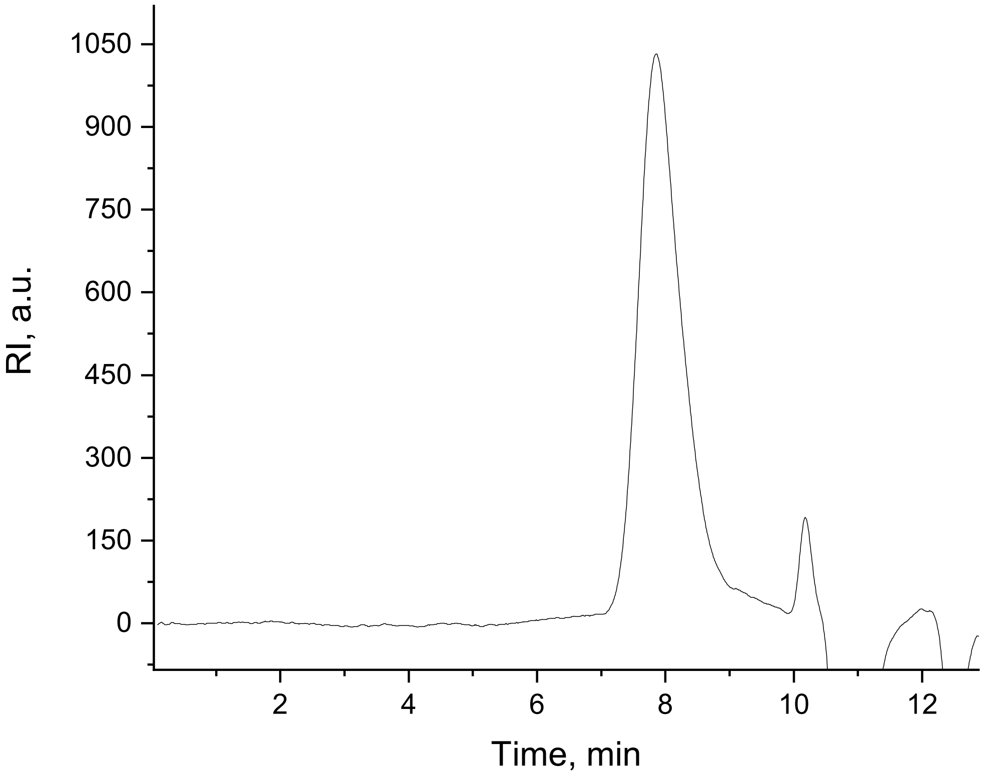

Figure S7. GPC data of the p-INT sample.

Non-covalent SP-3 for physical mixture preparation (p-MIX)

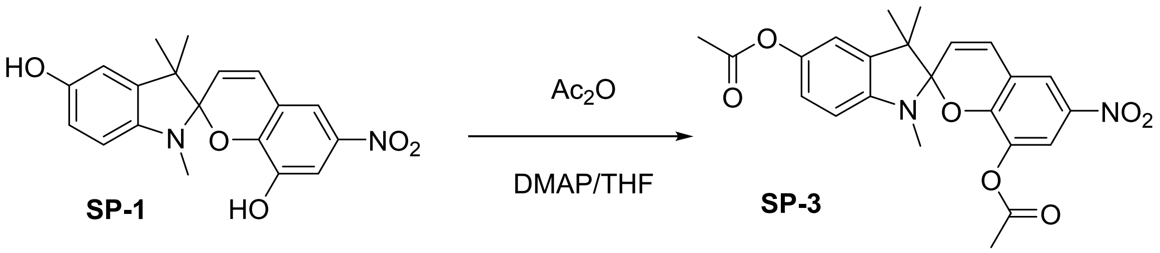

Spiropyran **SP-1** (706 mg, 2 mmol, 1 eq.) was loaded as solid in a 50 mL Schlenk tube followed by adding 560 mg of solid DMAP (4.9 mmol, 2.5 eq.). 35 mL of dry THF was added and the mixture was cooled down to 0°C with stirring under Ar. Acetic anhydride (0.410 mL, 4.3 mmol, 2.15 eq.) was added to the cold stirring mixture with a syringe and the reaction was allowed to proceed overnight warming up to room temperature in the course of the first 30 min. TLC indicated the formation of the reaction product and the consumption of starting materials; the reaction mixture was worked up as following: 60 mL of DCM was added to the reaction mixture, followed by 100 mL of water to partition the salts and the THF in the aqueous phase. The organic phase was further extracted with water (2x 50 mL) and dried with sodium sulfate, concentrated to dryness, and passed through an alumina plug (10 cm long, 5 cm in diameter) as a DCM solution in lieu of chromatography. The organic phase was then concentrated to dryness and triturated with 5 mL pentane to yield **SP-3** as a pale green solid. Y: 560 mg (70%)

NOTE: generally, double-esterified SP-1 derivatives did not require column chromatography and plug filtration sufficed. Typical R_f for these compounds on alumina were above 0.9 in DCM and the SP product could easily be identified as a pale yellow or colorless spot that rapidly changes color to purple under UV light (254 nm), and emitted orange luminescence under 365 nm light. The solutions of double-esterified SP-1 derivatives might appear green or pale blue.

NMR: ^{1}H NMR (400 MHz, $CDCl_3$) δ 7.94 (d, *J* = 2.6 Hz, 1H), 7.83 (d, *J* = 2.6 Hz, 1H), 6.98 (d, *J* = 10.4 Hz, 1H), 6.86 (dd, *J* = 8.3, 2.3 Hz, 1H), 6.81 (d, *J* = 2.3 Hz, 1H), 6.49 (d, *J* = 8.3 Hz, 1H), 5.92 (d, *J* = 10.4 Hz, 1H), 2.64 (s, 3H), 2.28 (s, 3H), 1.81 (s, 3H), 1.26 (s, 3H), 1.22 (s, 3H).
^{13}C NMR (100 MHz, $CDCl_3$) δ 170.21, 168.68, 151.01, 145.40, 144.40, 140.26, 137.89, 137.56, 128.71, 120.86, 120.35, 120.11, 119.41, 119.13, 115.50, 107.84, 107.61, 51.84, 28.96, 25.43, 21.21, 19.65, 19.55.
LC-MS: SP+H^+ 439.20 (Calc. 439.14) IMPORTANT: SP shows high affinity to the C18 column and elutes as two peaks in MeCN-water-formic acid. Total integration of TIC does not reveal any impurities except for target compound.

The physical mixture **p-MIX** was prepared by dissolving 5 mg SP-3 and 200 mg solid PMMA in chloroform, and cast as described above. Blank PMMA (26.85 kDa, PDI 2.02, DMA: T_g = 101.6°C

(1Hz)) was prepared using 2-cyano-2-propyl dodecyl trithiocarbonate RAFT agent in a procedure otherwise identical to that used for p-INT.

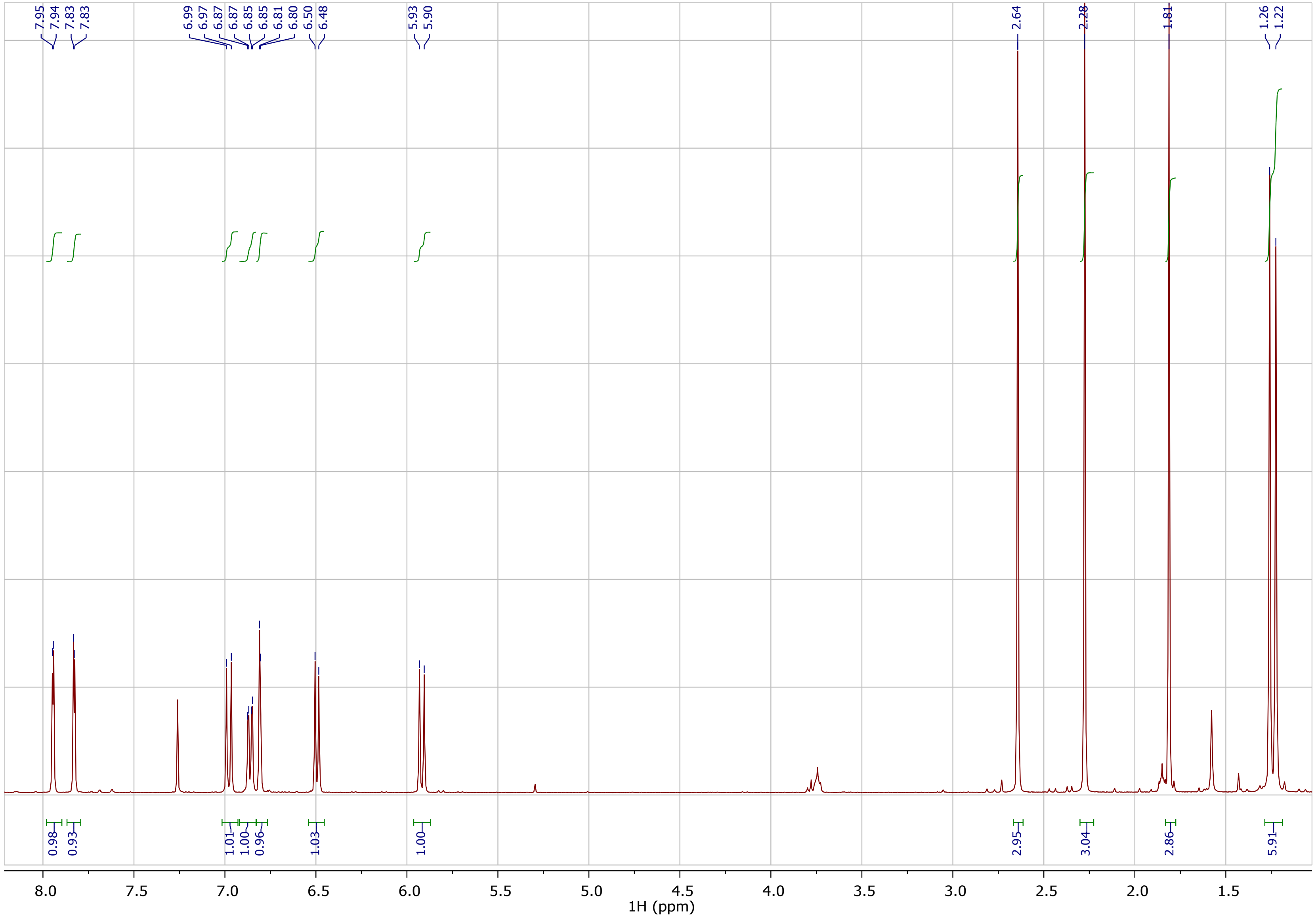

Figure S8. ^{1}H spectrum of SP-3 ($CDCl_3$).

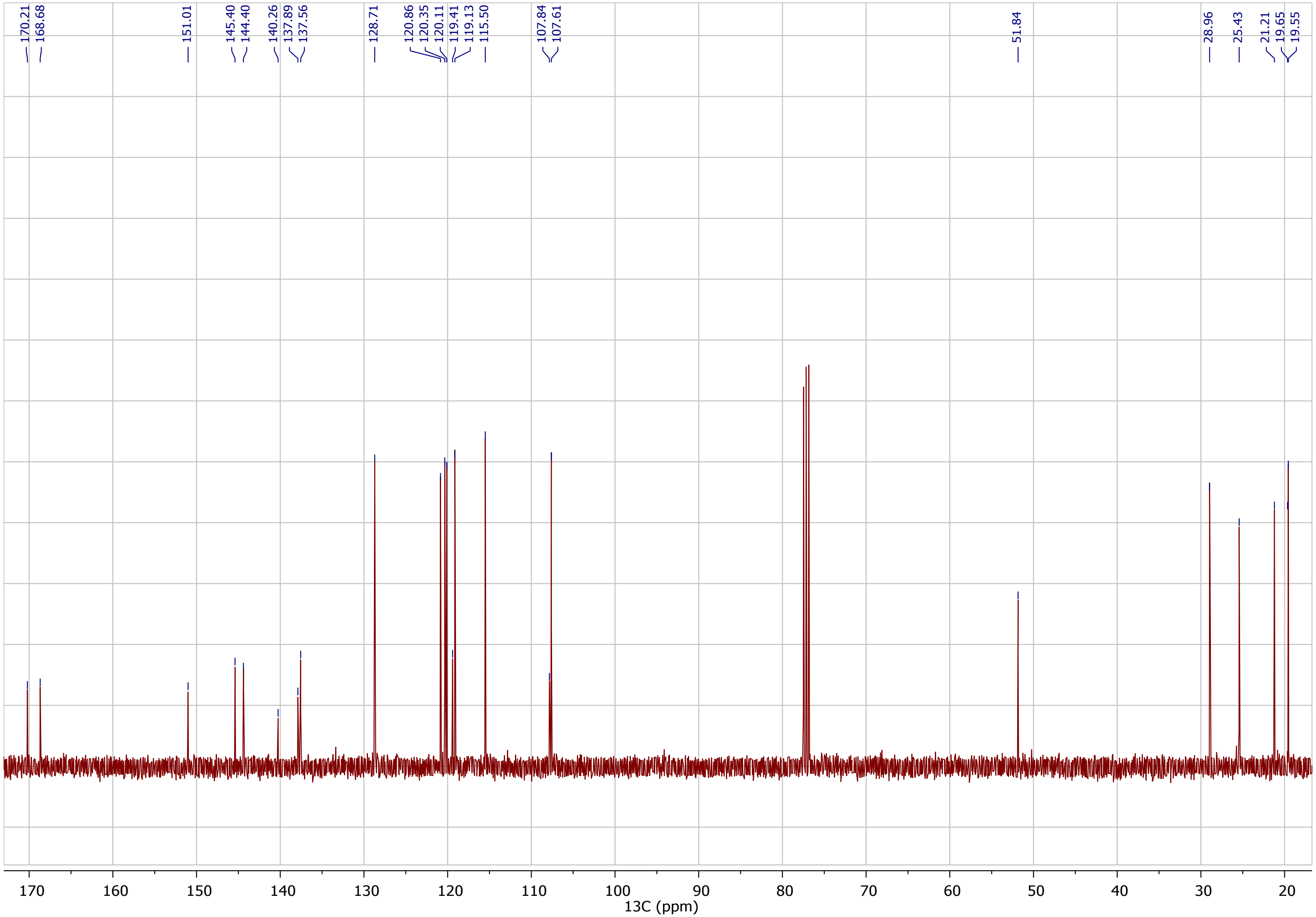

Figure S9. ^{13}C spectrum of SP-3 ($CDCl_3$).

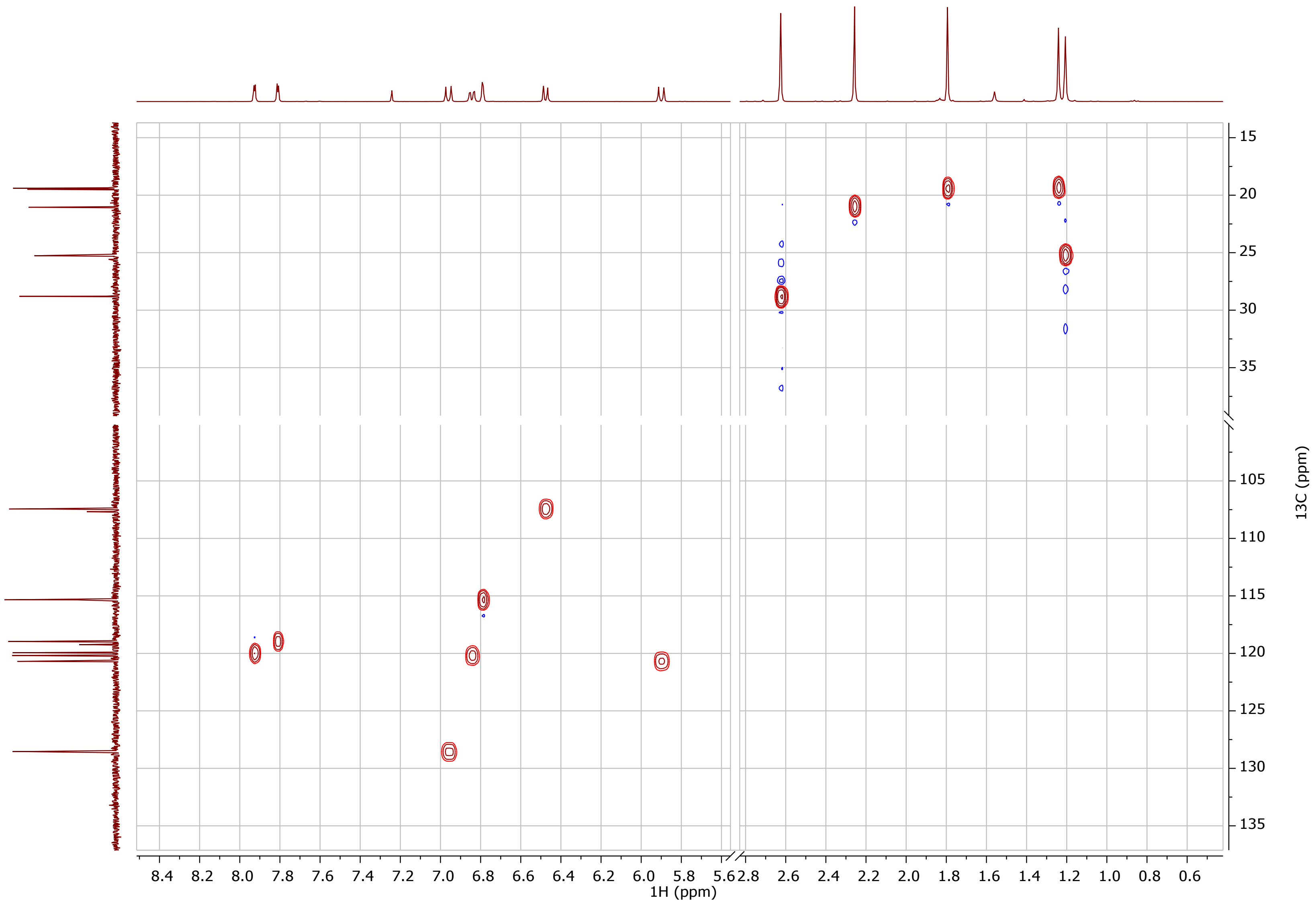

Figure S10. gHSQC spectrum of SP-3 ($CDCl_3$).

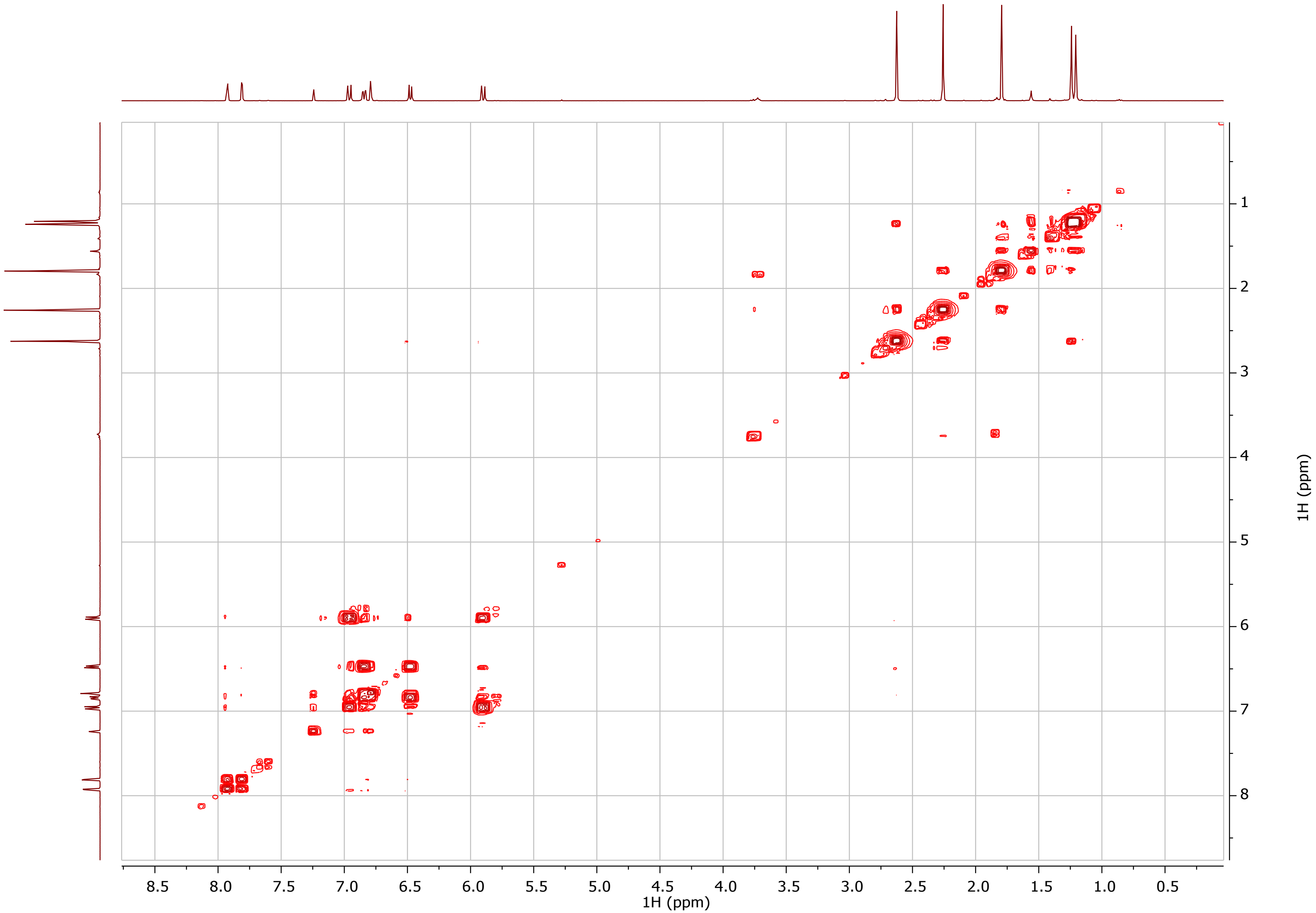

Figure S11. gCOSY spectrum of SP-3 ($CDCl_3$).

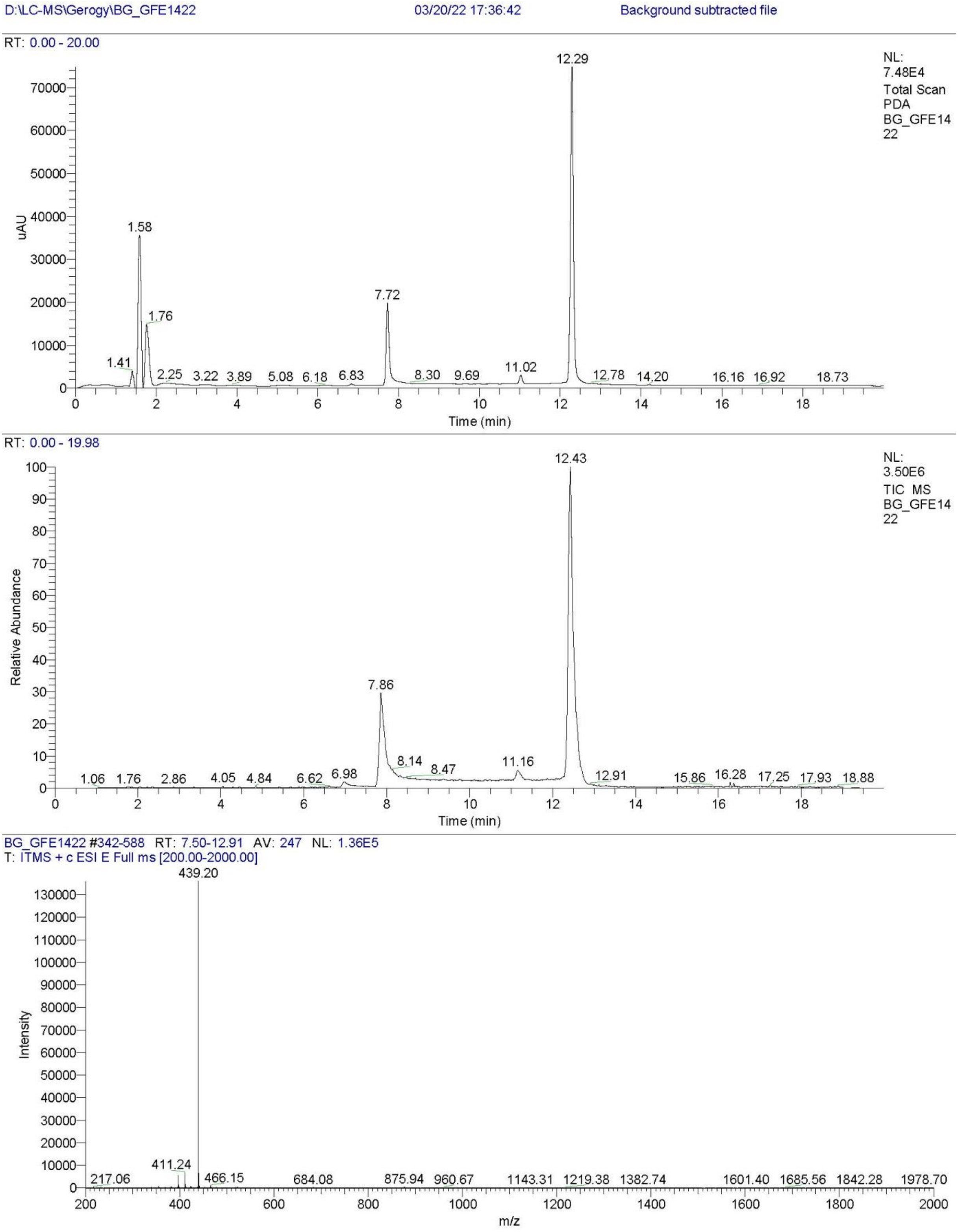

Figure S12. LC-MS data for SP-3.

Monofunctional SP-4

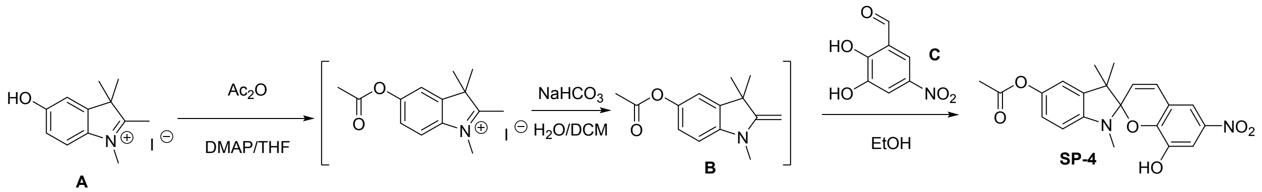

Compound **A**, an intermediate in SP-1 preparation, was acetylated as following: under Argon, 3.2 g **A** (10.09 mmol, 1 eq.) was suspended in 70 mL dry THF and treated with solid DMAP (3.6 g, 3 eq.) and acetic anhydride (2.88 mL, 3 eq.). The mixture was stirred at room temperature for 1 h and warmed up to 50°C for 1 h to complete the reaction. Upon completion, the mixture was cooled down to 10°C, DCM was added (150 mL), followed by the addition of 100 mL water. The organic phases were separated, extracted twice with saturated aqueous sodium bicarbonate, and dried with sodium sulfate. Upon filtration it was noted that the solution that dried out on a paper filter rim has left behind an intense pink coloration that indicated base-assisted rearrangement of the neutral product **B** instead of quaternary salt. This was confirmed by NMR spectroscopy and the filtrate was quickly purged with Argon flow and evaporated to dryness yielding 1.55 g of **B** that was used without intermediate purification.

^{1}H NMR (400 MHz, DMSO-d_6) δ 6.94 (d, J = 2.3 Hz, 1H), 6.81 (dd, J = 8.4, 2.4 Hz, 1H), 6.63 (d, J = 8.4 Hz, 1H), 3.86 (s, 2H), 3.00 (s, 3H), 2.21 (s, 3H), 1.27 (s, 6H).

SP-4:

To the crude **B** obtained above (1.55 g, 6.7 mmol) 1.227 g **C** (1 eq., prepared as intermediate in SP-1 synthesis) was added under Argon flow, together with 40 mL ethanol. The solution was then degassed and refluxed at 85°C for 3 h. Afterwards, the reaction mixture was cooled down to -20°C (2 h), filtered in air on a porous glass frit, and washed with three portions of cold ethanol, ether, and pentane. The resulting purple powder was vacuum dried to obtain **SP-4**. Y: 2.349 g (88.5 %)

^{1}H NMR (400 MHz, $CDCl_3$) δ 7.69 (d, J = 2.6 Hz, 1H), 7.64 (d, J = 2.6 Hz, 1H), 6.95 – 6.87 (m, 2H), 6.82 (d, J = 2.3 Hz, 1H), 6.52 (d, J = 8.3 Hz, 1H), 5.85 (d, J = 10.4 Hz, 1H), 2.74 (s, 3H), 2.29 (s, 3H), 1.27 (s, 3H), 1.21 (s, 3H).
^{13}C NMR (100 MHz, $CDCl_3$) δ 170.44, 146.49, 145.31, 144.45, 143.64, 141.34, 137.20, 128.85, 121.30, 120.71, 118.28, 115.95, 114.43, 111.53, 108.02, 107.39, 52.44, 29.15, 25.93, 21.24, 19.77.
LC-MS: SP+H^+ 398.19 (Calc. 397.14) IMPORTANT: SP shows high affinity to the C18 column and elutes as two peaks in MeCN-water-formic acid. Total integration of TIC does not reveal any impurities except for target compound.

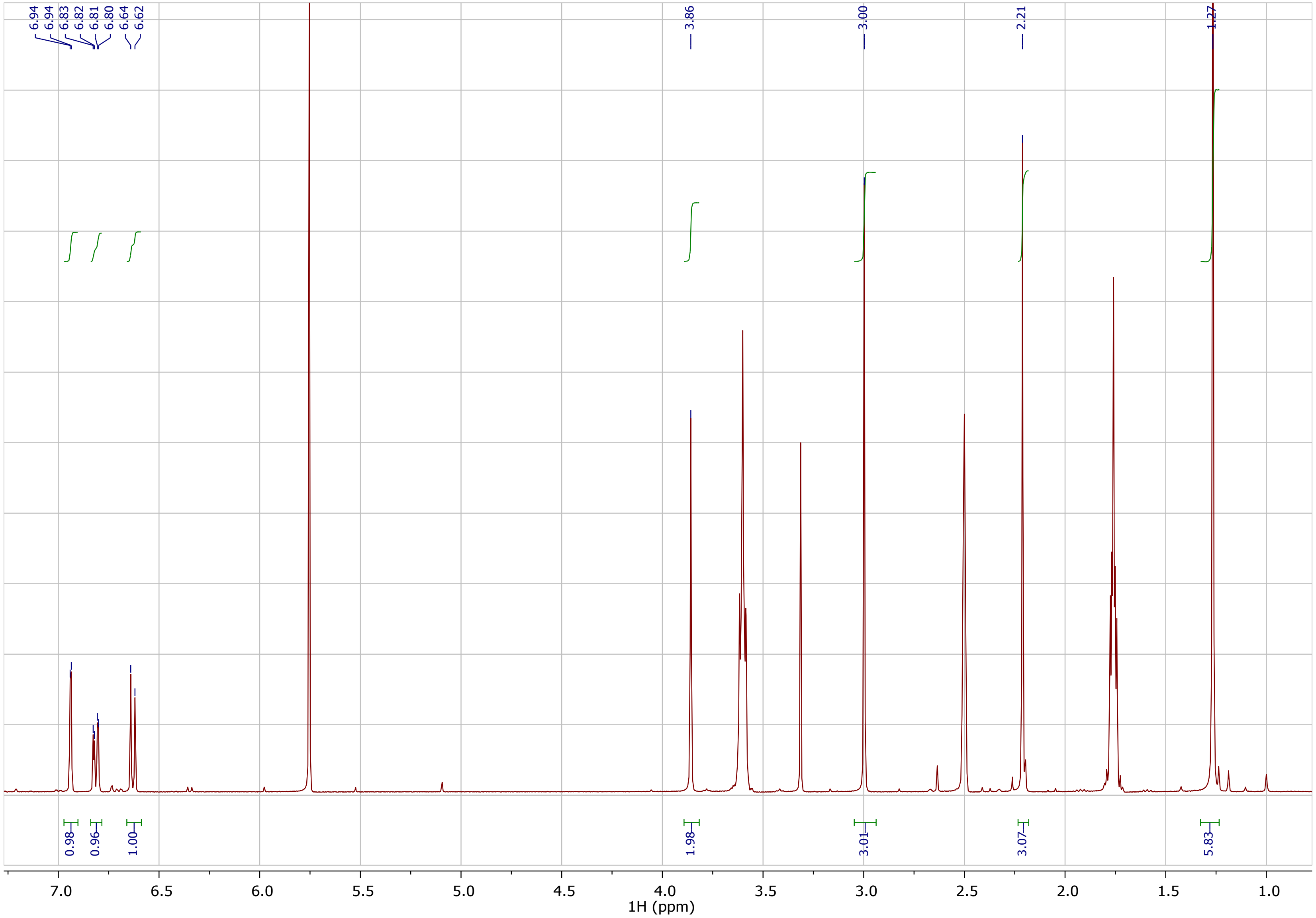

Figure S13. ^{1}H spectrum of intermediate **B** of SP-4 synthesis (DMSO-d_6).

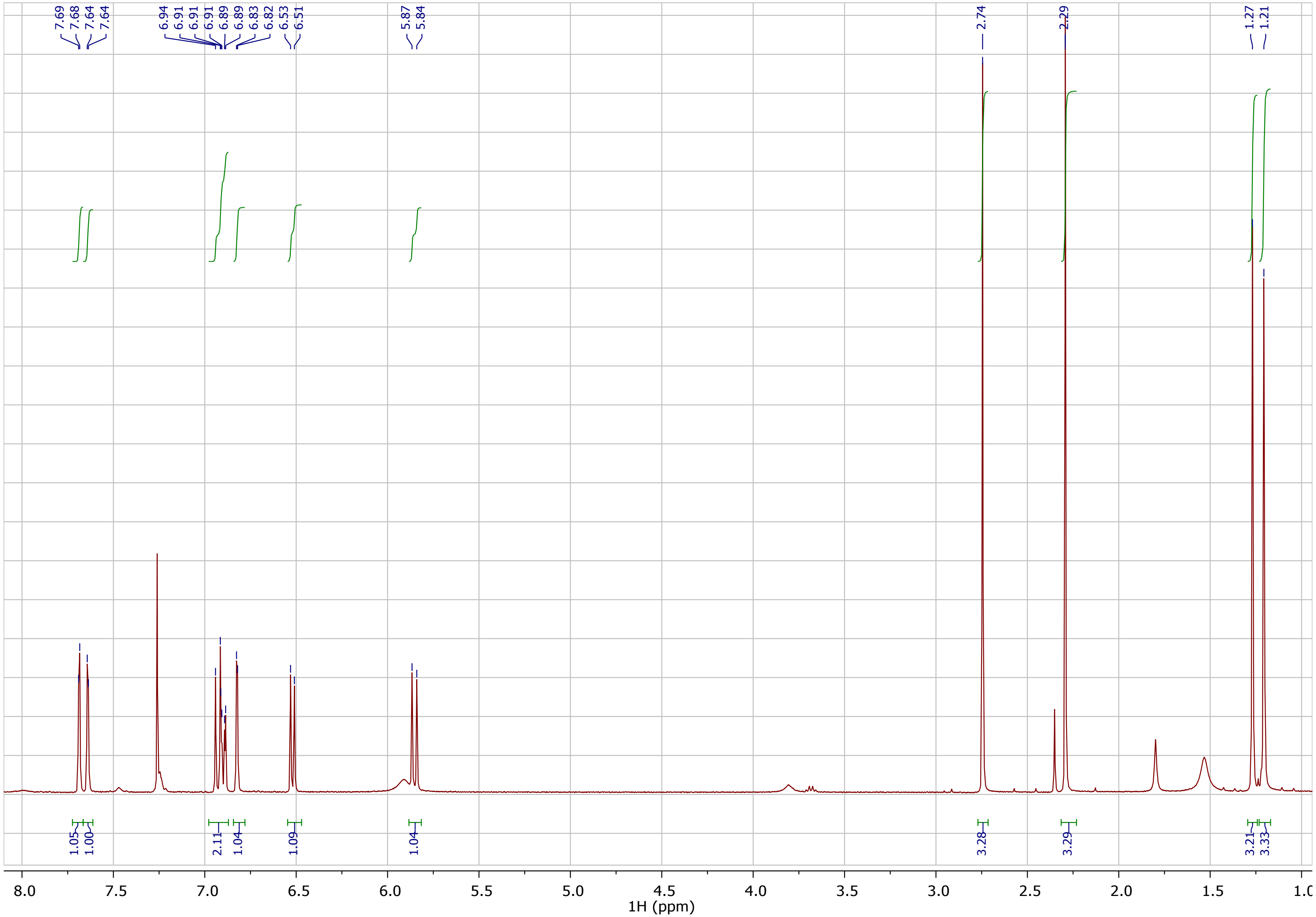

Figure S14. ^{1}H spectrum of SP-4 ($CDCl_3$).

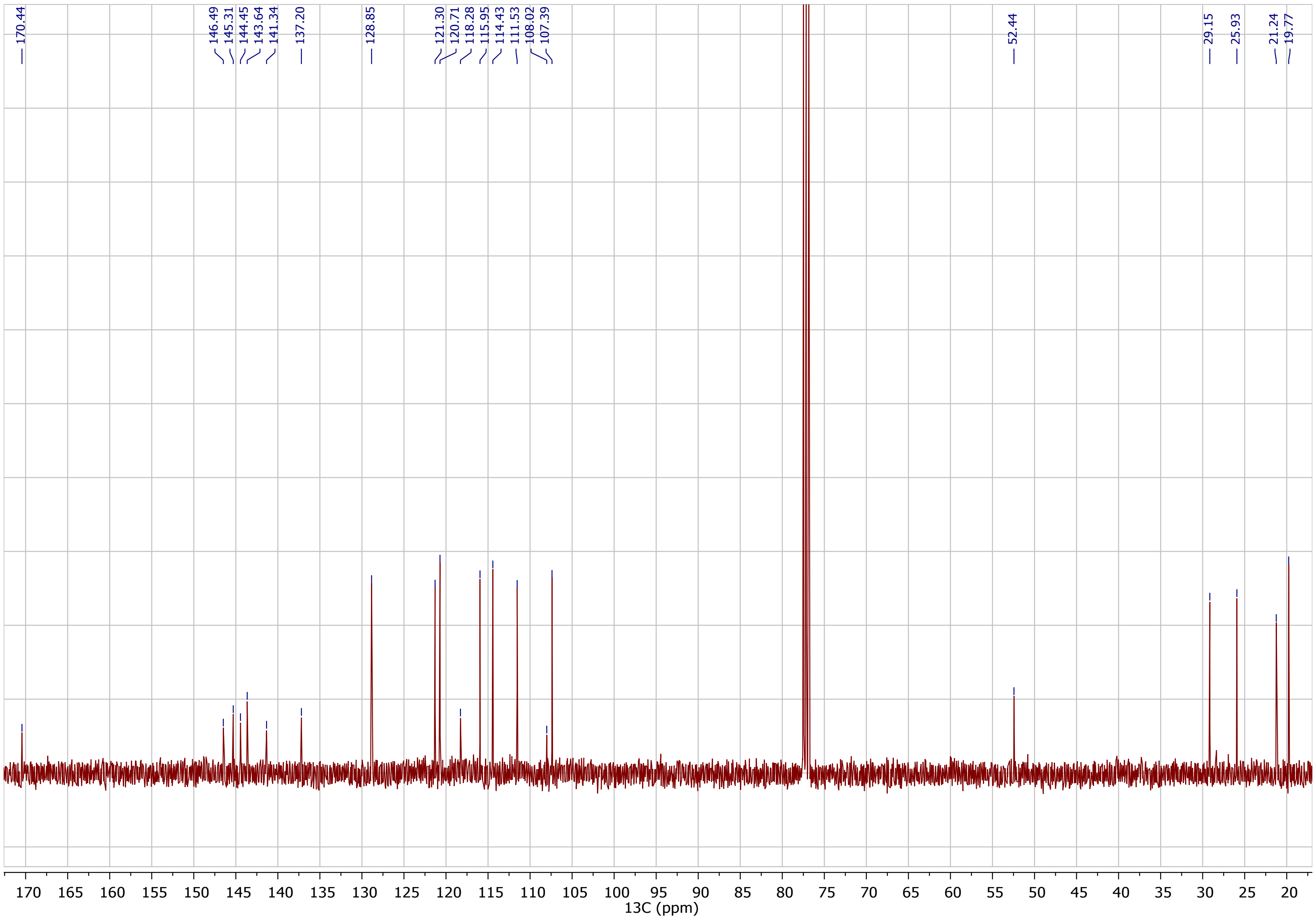

Figure S15. ^{13}C spectrum of SP-4 ($CDCl_3$).

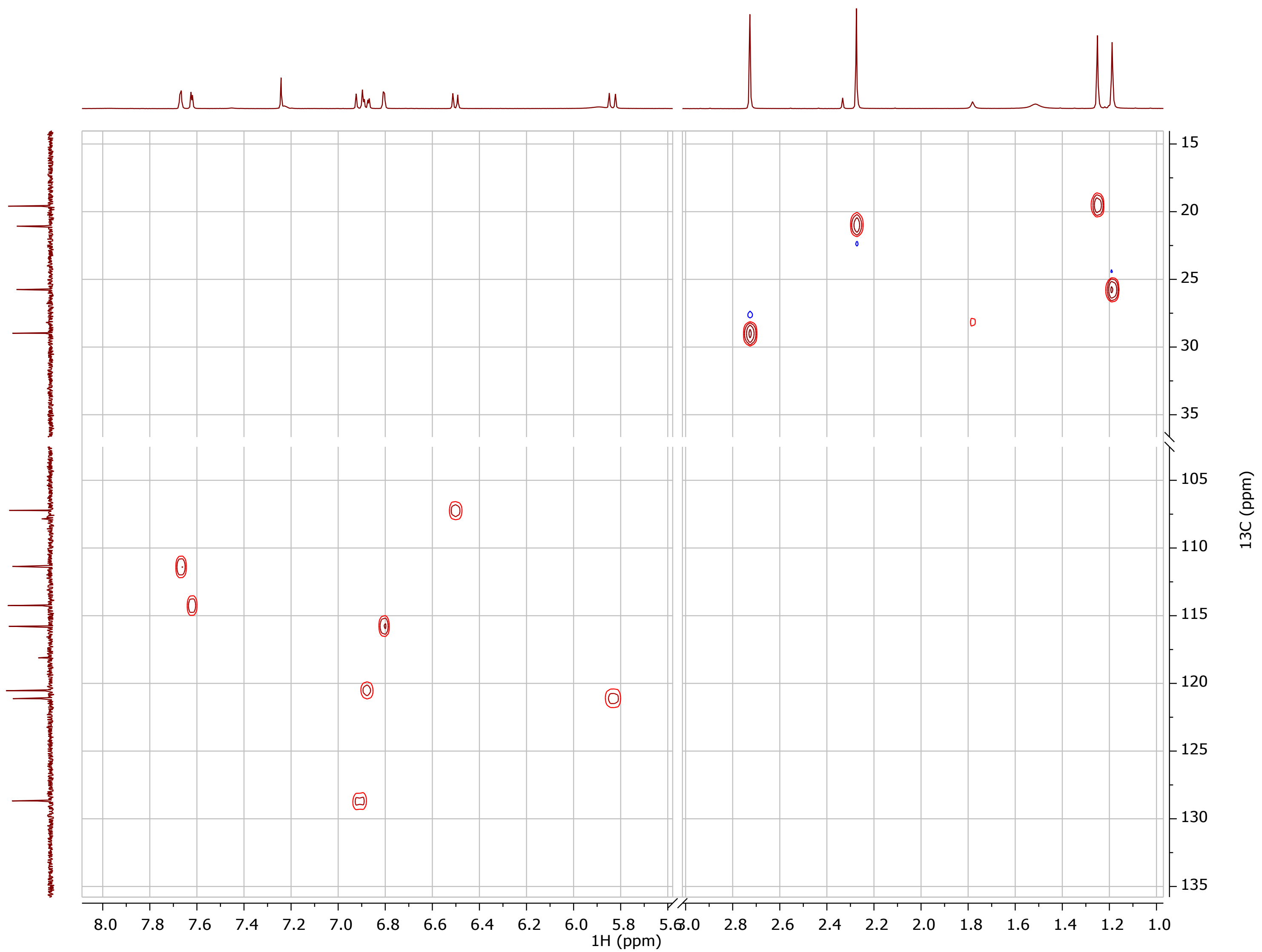

Figure S16. gHSQC spectrum of SP-4 ($CDCl_3$).

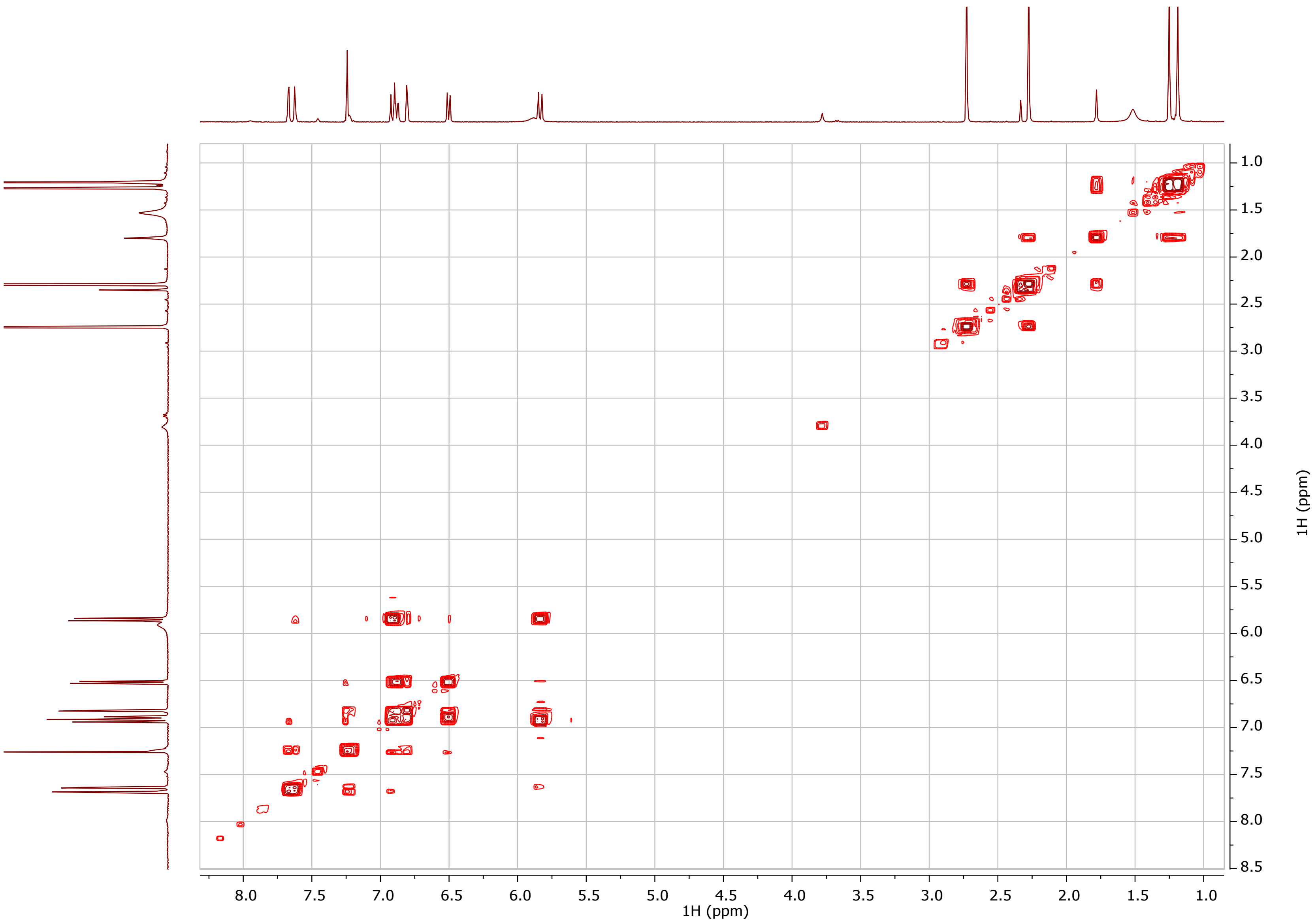

Figure S17. gCOSY spectrum of SP-4 ($CDCl_3$).

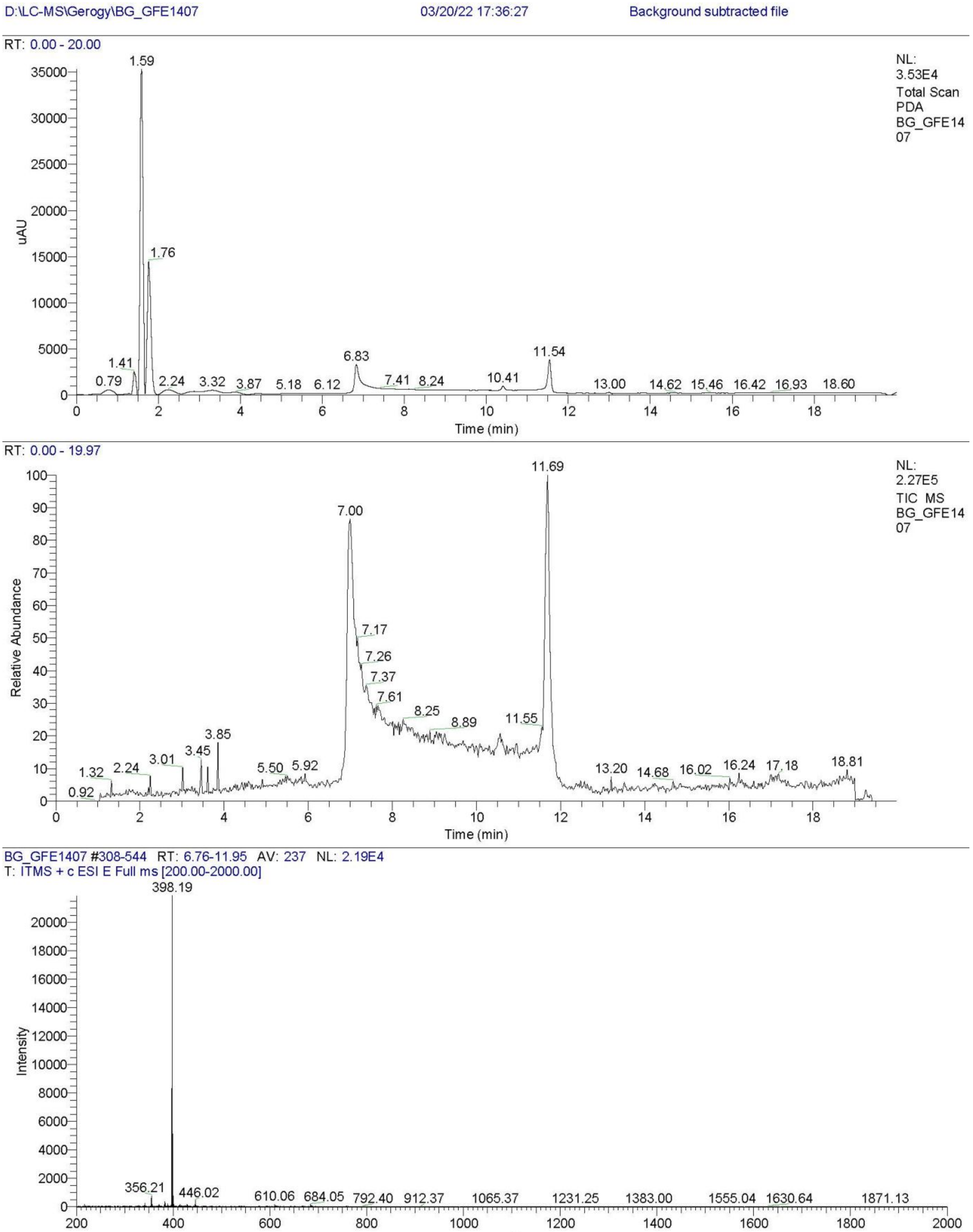

Figure S18. LC-MS data for SP-4.

Chain end PMMA (p-CE) and monofunctional CTA SP-5

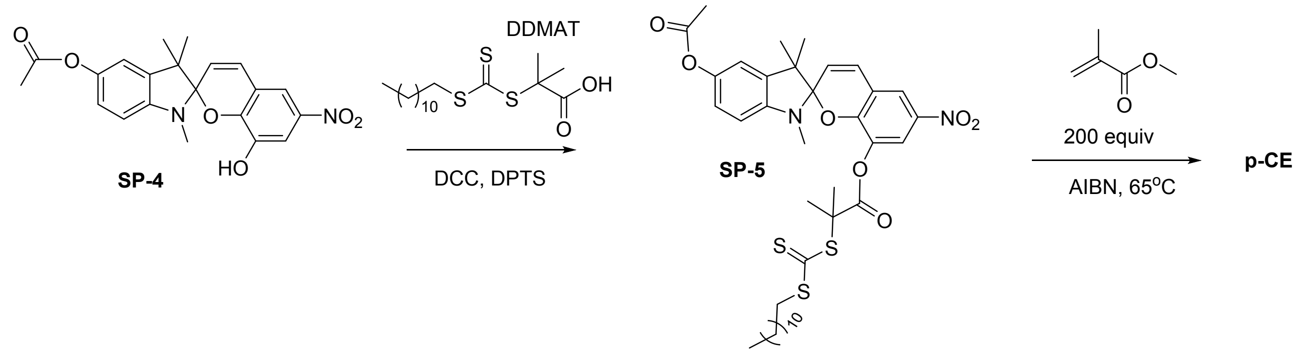

SP-4 (321 mg, 0.81 mmol, 1 eq) was converted in a procedure similar to that used for bifunctional SP-2. Loadings used were as following: 309 mg of DDMAT (0.849 mmol, 1.05 eq.), 236 mg of DPTS (0.80 mmol, ca 1 eq), and 215 mg of DCC (1.048 mmol, 1.3 eq). The workup was identical to SP-2. The resulting green-colored oil slowly solidifies at 4 °C. Y:420 mg (ca 70%).

^{1}H NMR (400 MHz, $CDCl_3$) δ 7.94 (d, J = 2.6 Hz, 1H), 7.72 (d, J = 2.6 Hz, 1H), 6.98 (d, J = 10.4 Hz, 1H), 6.83 (dd, J = 8.3, 2.3 Hz, 1H), 6.77 (d, J = 2.3 Hz, 1H), 6.47 (d, J = 8.3 Hz, 1H), 5.91 (d, J = 10.4 Hz, 1H), 3.31 (t, J = 7.4 Hz, 2H), 2.67 (s, 3H), 2.27 (s, 3H), 1.67 (m, 2H), 1.33 (m, 2H), 1.35 (s, 3H), 1.25 (d, J = 4.1 Hz, 18H), 1.21 (s, 3H), 1.18 (s, 3H), 0.87 (t, J = 6.8 Hz, 3H).
^{13}C NMR (100 MHz, $CDCl_3$) δ 221.76, 170.30, 169.98, 151.38, 145.43, 144.58, 140.28, 137.57, 137.43, 128.72, 120.90, 120.48, 120.32, 119.75, 119.44, 115.62, 107.67, 107.44, 55.51, 51.87, 37.28, 32.03, 29.75, 29.73, 29.66, 29.56, 29.45, 29.22, 29.07, 28.87, 28.02, 25.96, 24.59, 24.48, 22.81, 21.20, 19.49, 14.24.
LC-MS: SP+H^+ 742.88 (Calc. 743.28) IMPORTANT: SP shows high affinity to the C18 column and elutes as two peaks in MeCN-water-formic acid. Total integration of TIC does not reveal any impurities except for target compound.

Polymerization to p-CE:
Methyl methacrylate (0.3 mL, 2.8164 mmol, ca. 160 eq) and 0.3 mL benzene-d_6 were added to an NMR tube containing 13.2 mg of SP-5 (0.0177 mmol, 1 eq.) and 0.5 mg of AIBN (0.003 mmol). The mixture was homogenized and set at 65°C overnight.

Workup according to general procedure
A 100 mg portion of dried polymer was analyzed using NMR spectroscopy and the solution in $CDCl_3$ was used directly for casting on glass cover slips. The cover slips were covered with a glass lid to prevent rapid evaporation and after 4 h the cover slips were dried in the vacuum oven at 65°C overnight (12-16 hours).

GPC: 35.26 kDa (PDI 1.60)

DMA: T_g = 97.9°C (1Hz)

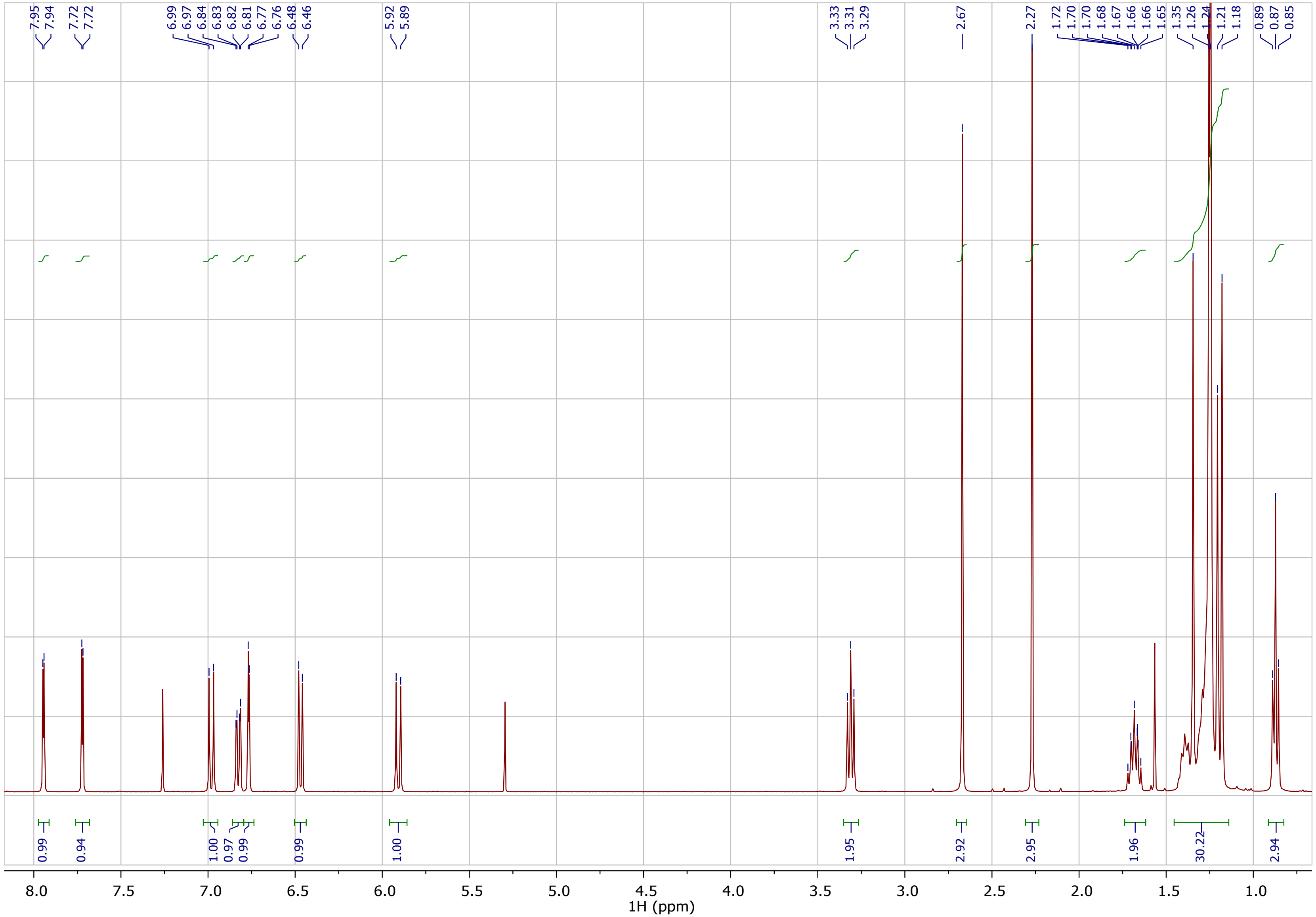

Figure S19. ^{1}H spectrum of SP-5 ($CDCl_3$).

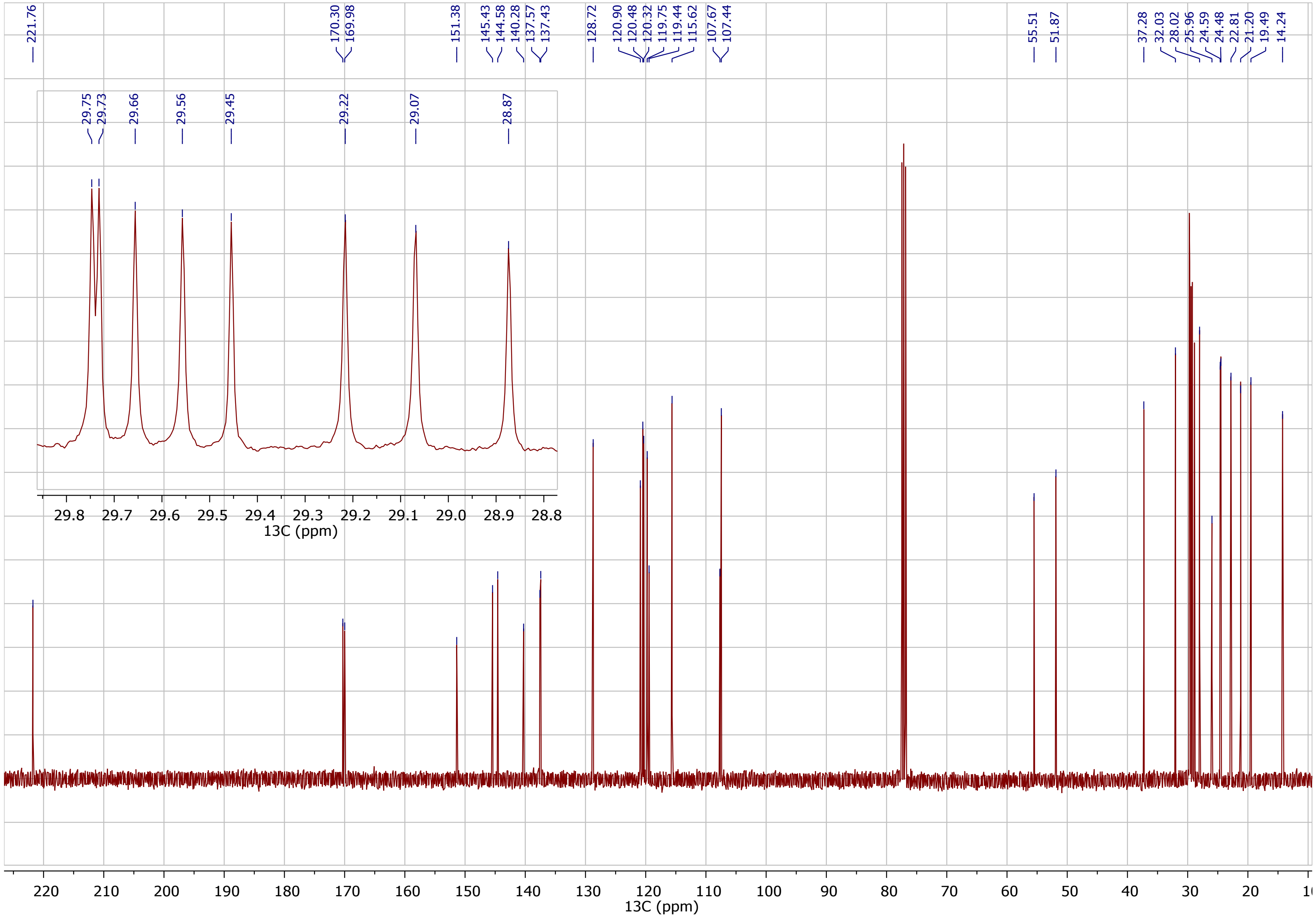

Figure S20. ^{13}C spectrum of SP-5 ($CDCl_3$).

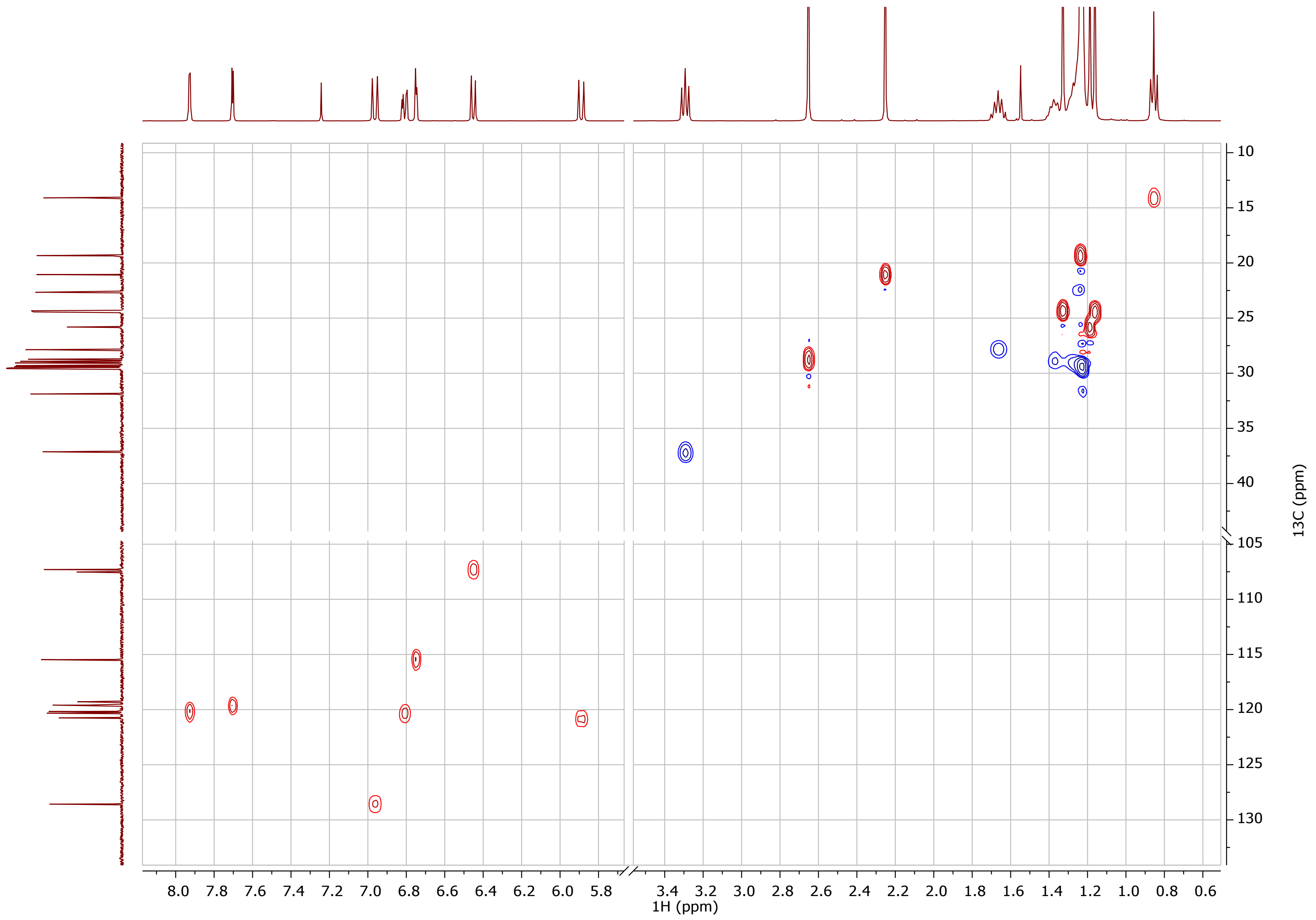

Figure S21. gHSQC spectrum of SP-5 ($CDCl_3$).

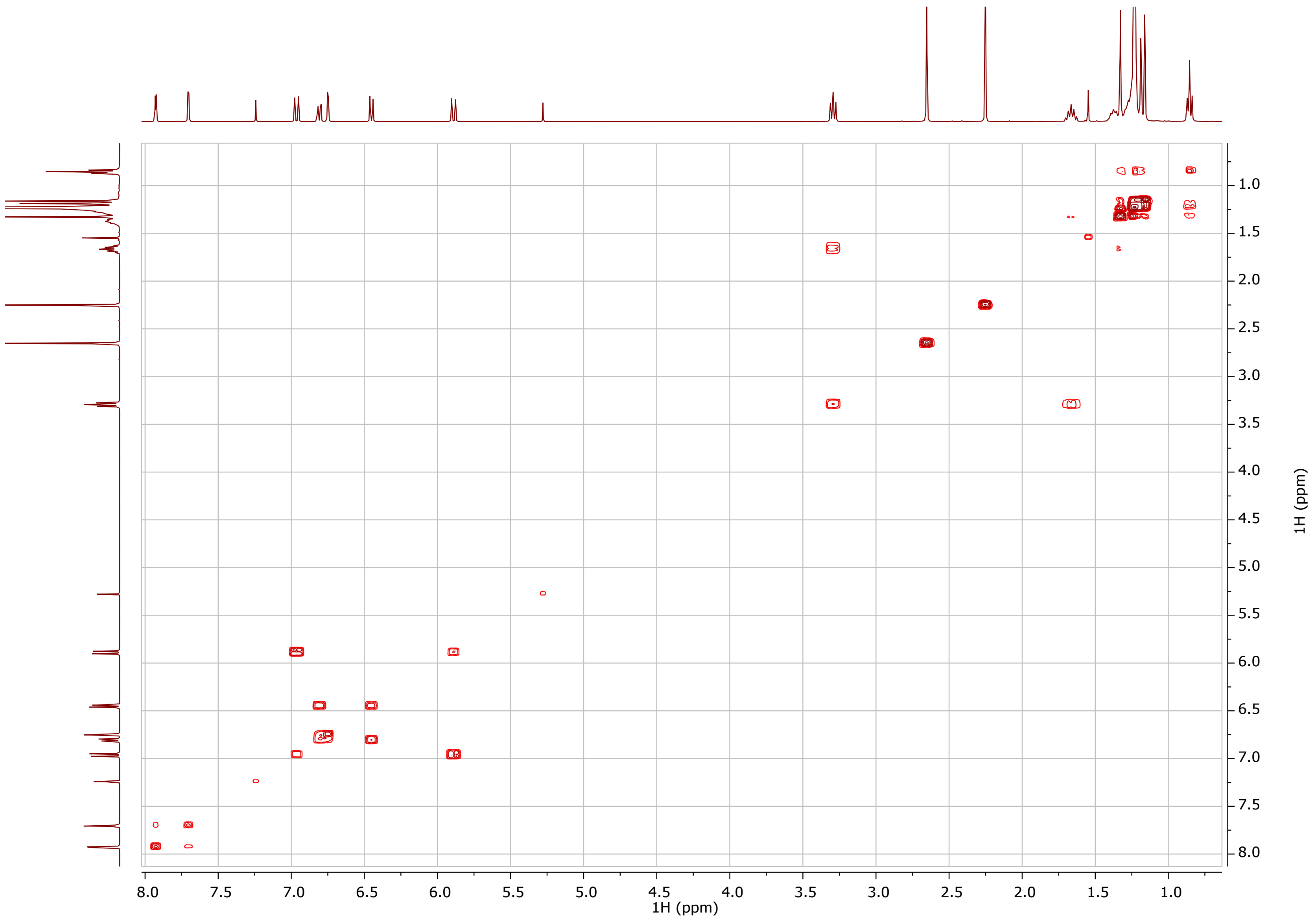

Figure S22. gCOSY spectrum of SP-5 ($CDCl_3$).

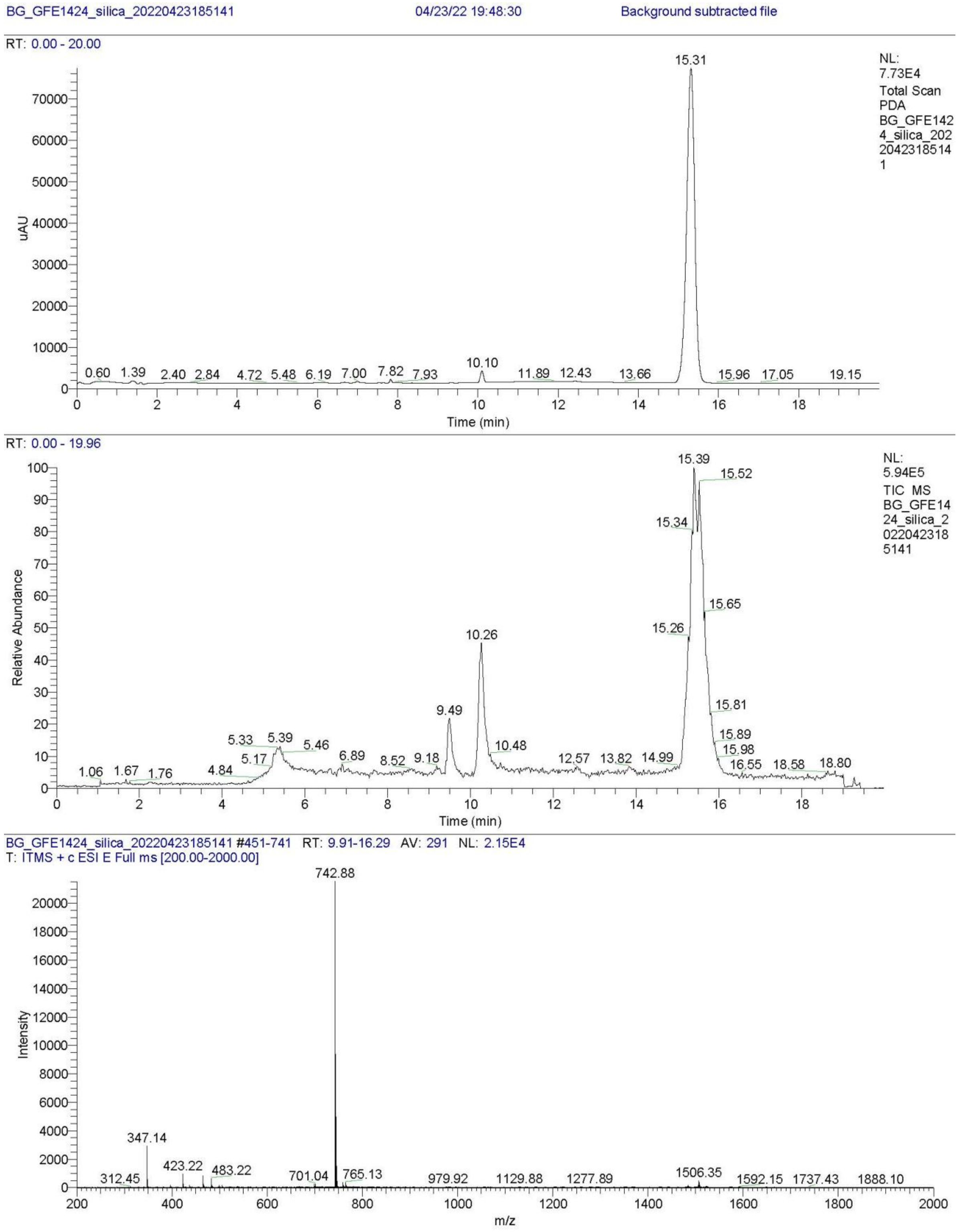

Figure S23. LC-MS data for SP-5.

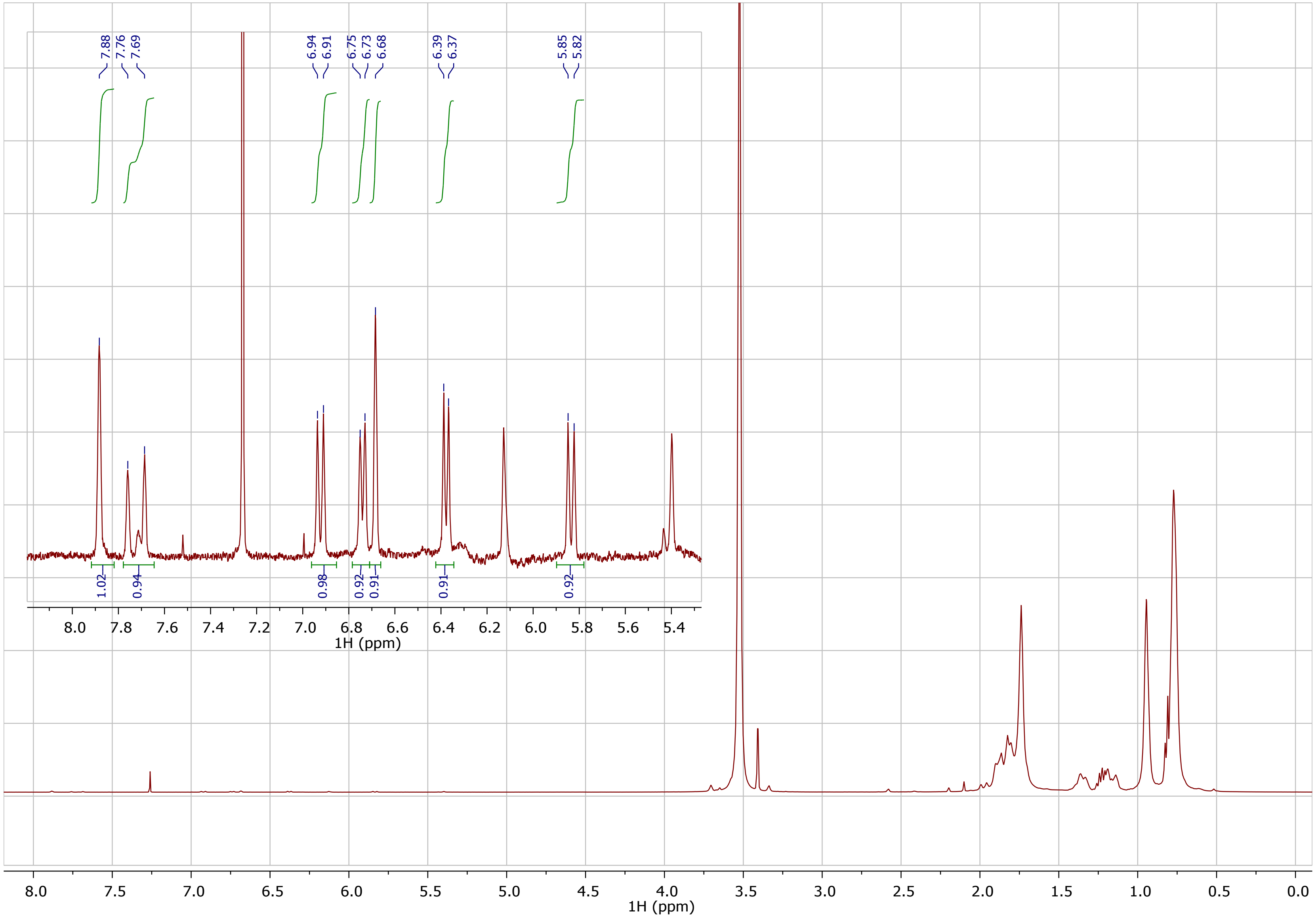

Figure S24. ^{1}H NMR spectrum of p-CE ($CDCl_3$).

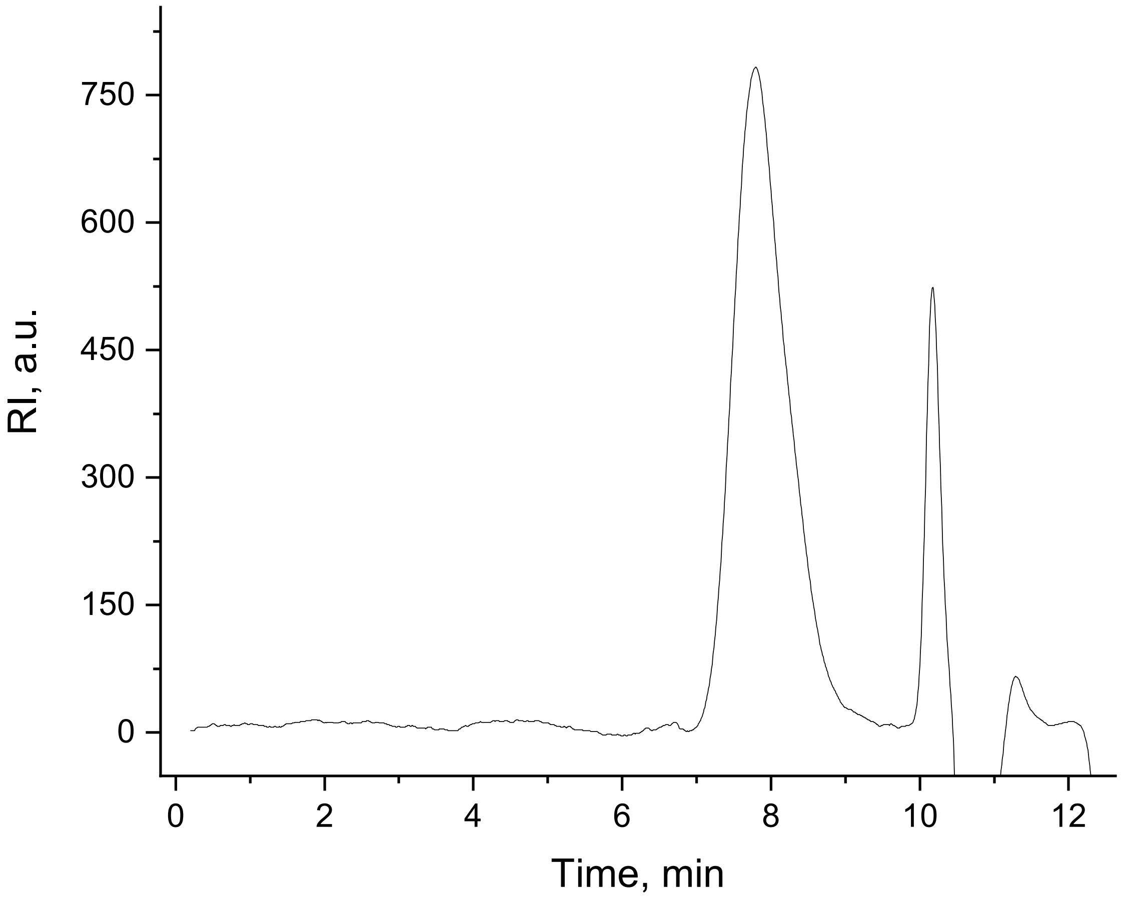

Figure S25. GPC data for p-CE.

Side chain PMMA (p-SC) and monofunctional monomer SP-6

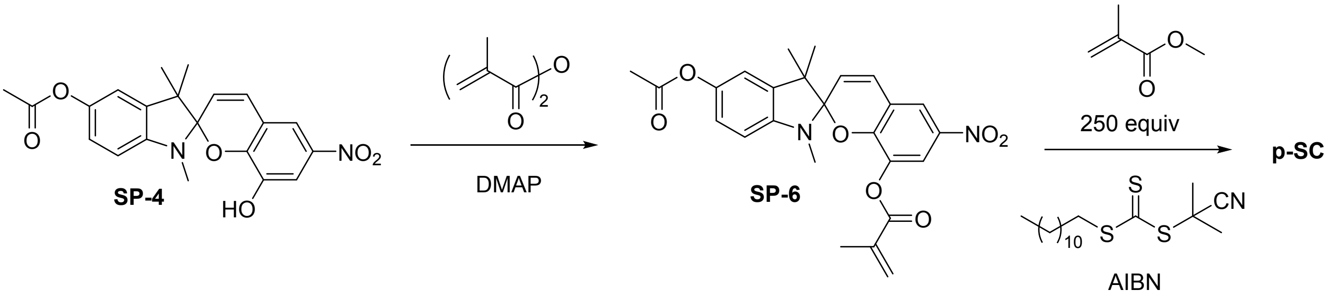

Spiropyran **SP-4** (990 mg, 2.5 mmol, 1 eq.), loaded as a solid in a 50 mL Schlenk tube, was followed by the addition of 610 mg of solid DMAP (5 mmol, 2 eq.). 40 mL of dry THF was added and the mixture was cooled down to 0°C with stirring under Argon. Methacrylic anhydride (0.446 mL, 3 mmol, 1.2 eq., DISTILLED BEFORE USE) was added to the cold stirring mixture with a syringe and the reaction was allowed to warm up overnight to room temperature in the course of the first 30 min. TLC indicated the formation of the reaction product (R_f ca. 0.7 in DCM on alumina) and consumption of the starting materials; the reaction mixture was worked up as following:100 mL DCM was added to the reaction mixture and further extracted with water (2x100 mL) to remove protonated DMAP, dried with sodium sulfate, concentrated to dryness, and passed through an alumina plug (10 cm long, 5 cm in diameter) as a DCM solution in lieu of chromatography. The organic phase was then concentrated to dryness and triturated with 5 mL pentane to yield **SP-6** as a pale green solid. Y: 950 mg (82%)

^{1}H NMR (400 MHz, $CDCl_3$) δ 7.95 (d, J = 2.6 Hz, 1H), 7.91 (d, J = 2.6 Hz, 1H), 6.98 (d, J = 10.4 Hz, 1H), 6.82 (dd, J = 8.3, 2.3 Hz, 1H), 6.74 (d, J = 2.3 Hz, 1H), 6.44 (d, J = 8.3 Hz, 1H), 5.99 – 5.79 (m, overlap of 2 X 1H), 5.48 (s, 1H), 2.63 (s, 3H), 2.27 (s, 3H), 1.67 (s, 3H), 1.23 (s, 3H), 1.20 (s, 3H).
^{13}C NMR (100 MHz, $CDCl_3$) δ 170.02, 165.11, 150.91, 145.24, 144.48, 140.25, 138.16, 137.38, 134.59, 128.59, 127.93, 120.92, 120.24, 119.93, 119.41, 119.21, 115.32, 107.45, 107.40, 51.76, 28.89, 25.63, 21.23, 19.61, 17.81.
LC-MS: SP+H^+ 465.22 (Calc. 465.16) IMPORTANT: SP shows high affinity to the C18 column and elutes as two peaks in MeCN-water-formic acid. Total integration of TIC does not reveal any impurities except for target compound.

Polymerization to p-SC

5 mg of SP-6 (10.7 µmol) was added to the NMR tube followed by adding 0.3 mL benzene d-6, 0.3 mL methyl methacrylate (263 eq), 7.4 mg 2-cyano-2-propyl dodecyl trithiocarbonate CTA (21.4 µmol, 2 eq. shown above), and 0.4 mg AIBN initiator. The reaction was set to 65°C overnight and worked-up as described above for other PMMA samples.

GPC: 8.35 kDa (PDI 1.28)
DMA: T_g = 112.6°C (1Hz)

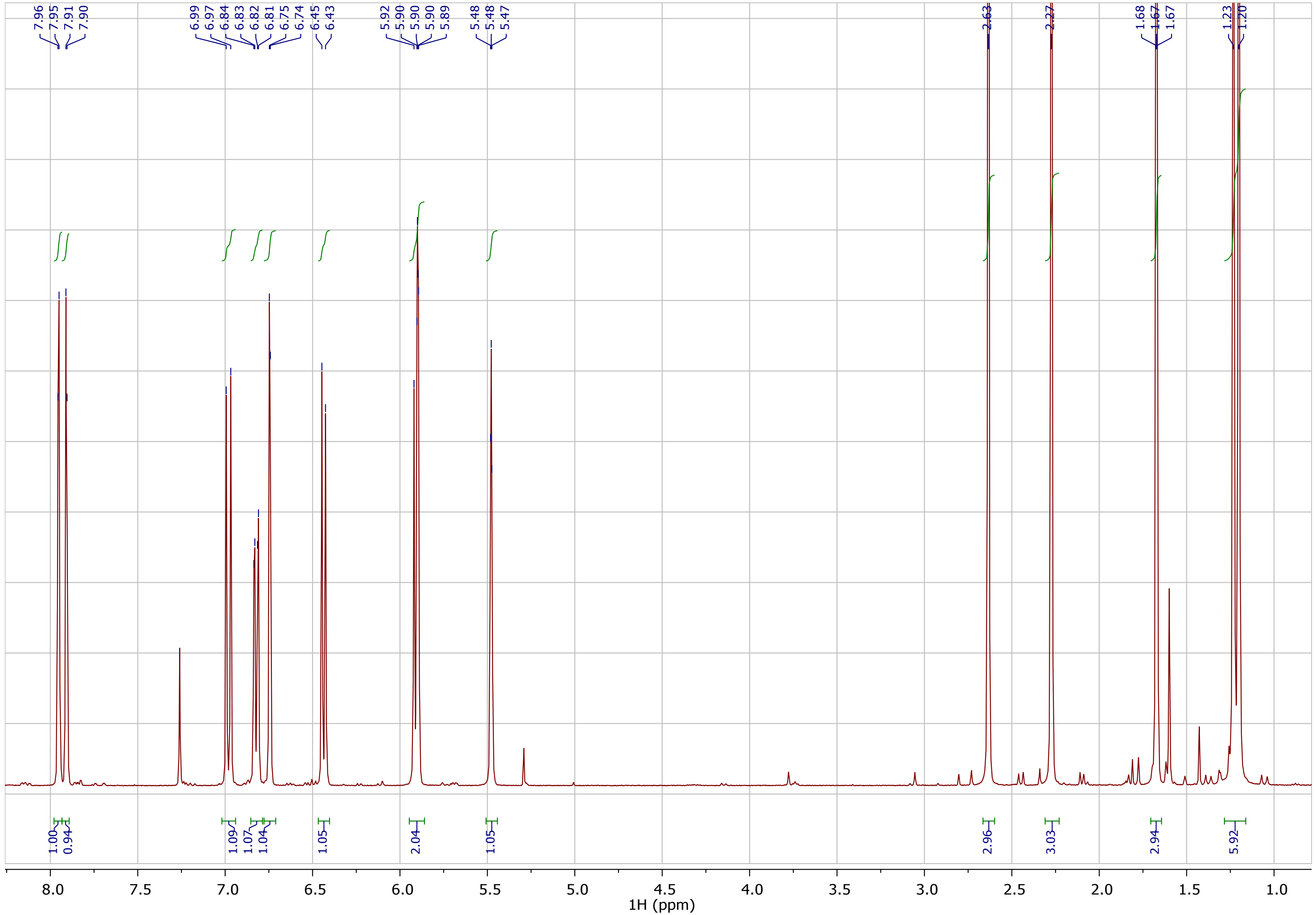

Figure S26. ^{1}H NMR spectrum of SP-6 ($CDCl_3$).

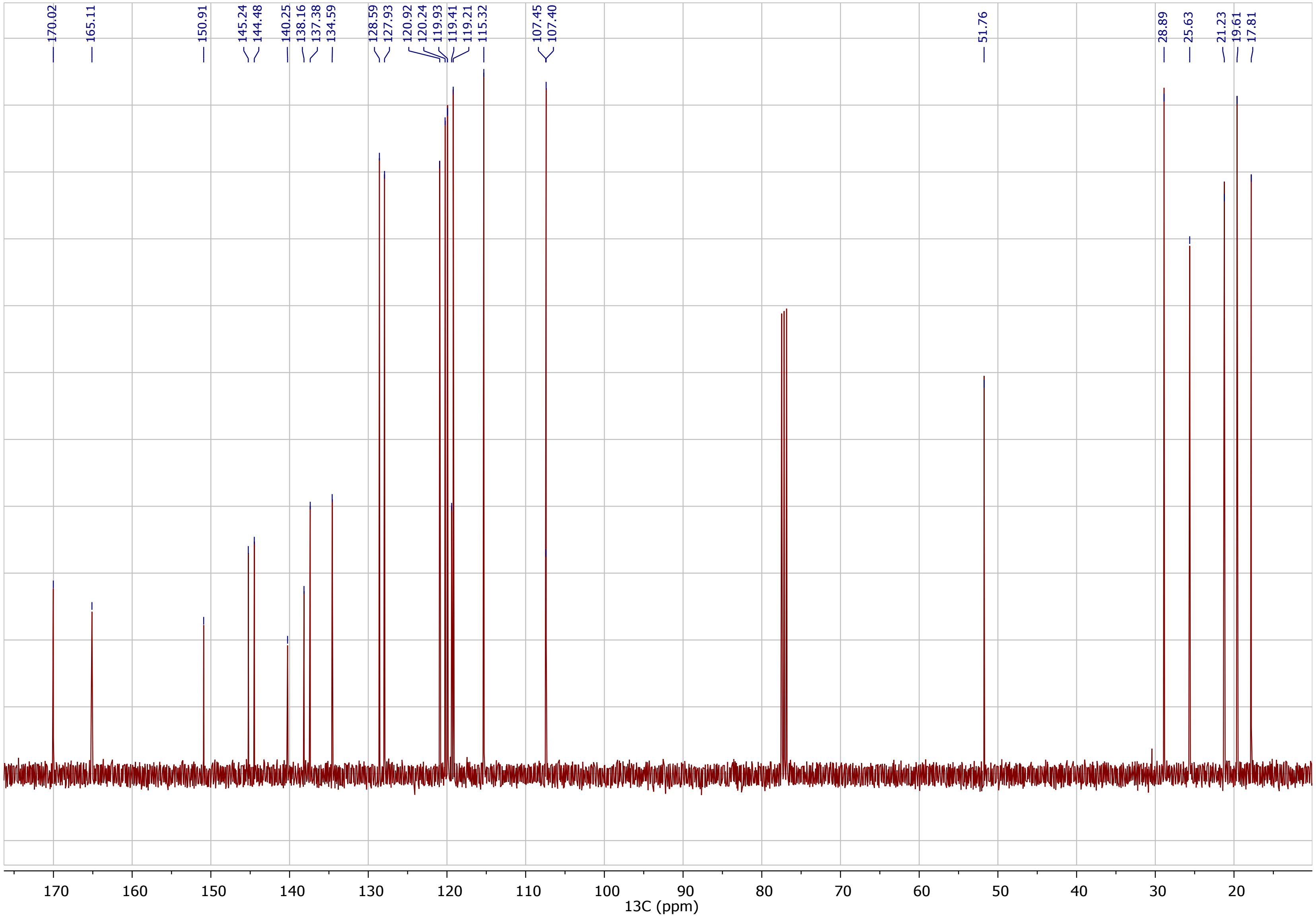

Figure S27. ^{13}C NMR spectrum of SP-6 ($CDCl_3$).

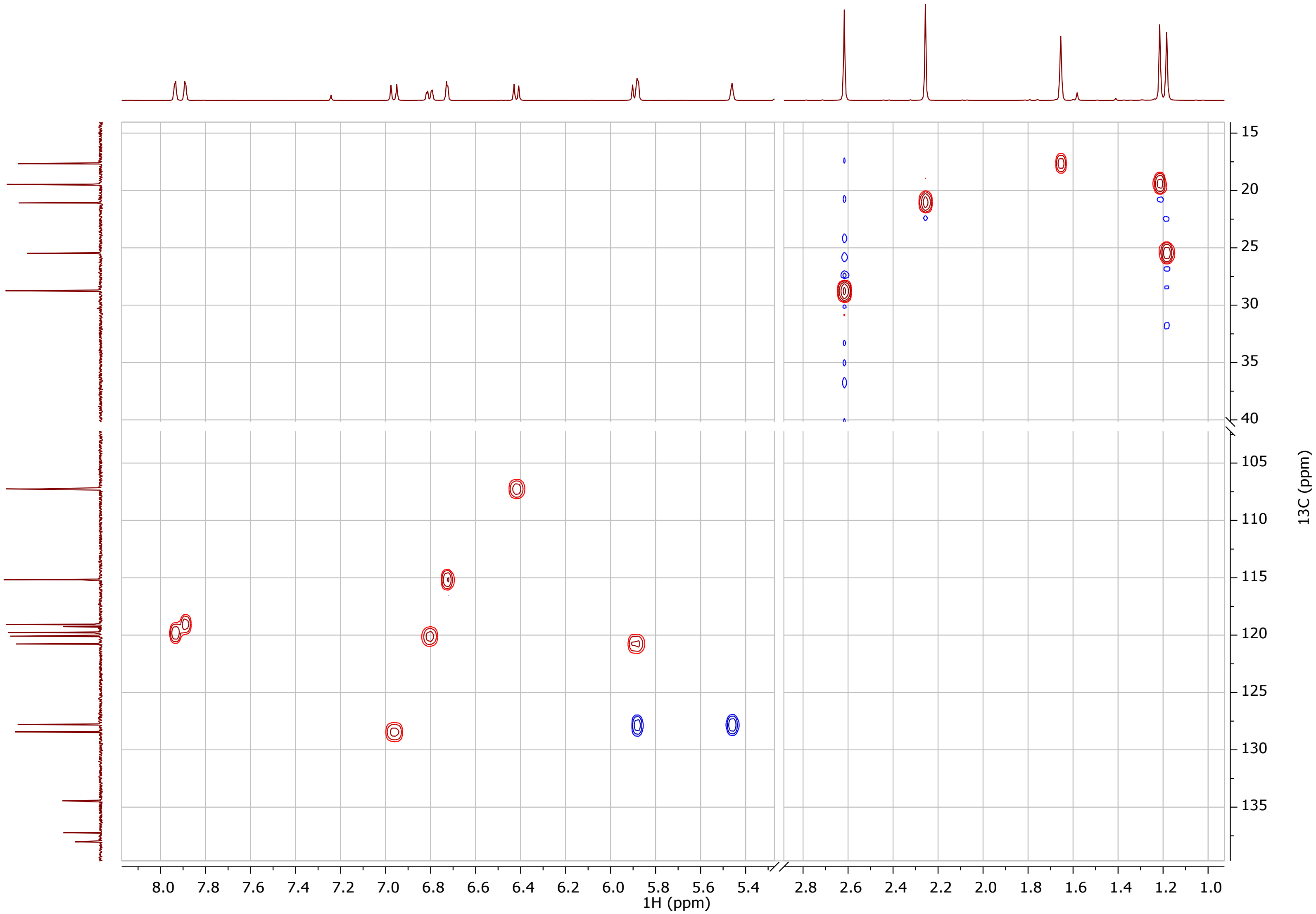

Figure S28. gHSQC spectrum of SP-6 ($CDCl_3$).

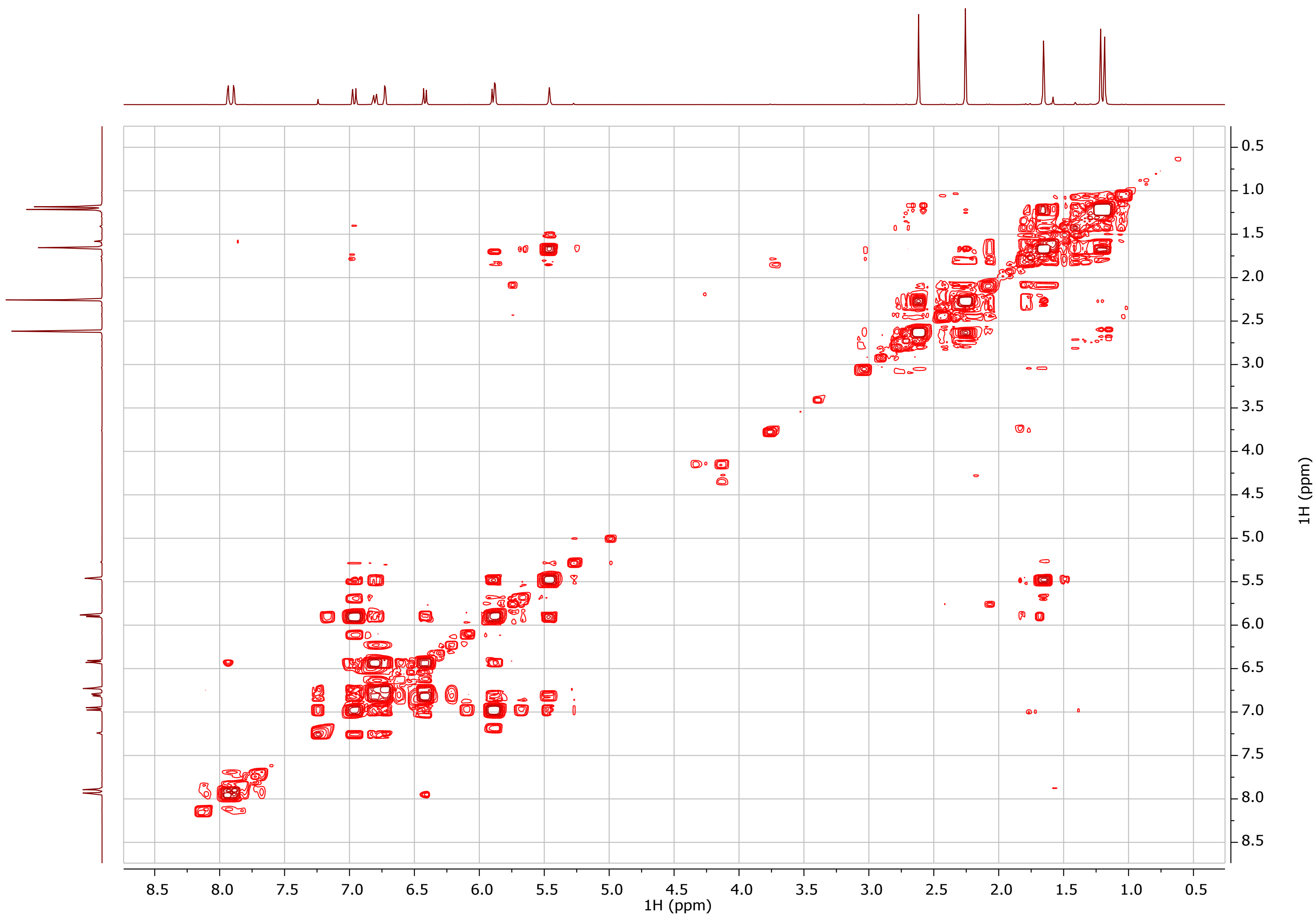

Figure S29. gCOSY spectrum of SP-6 ($CDCl_3$).

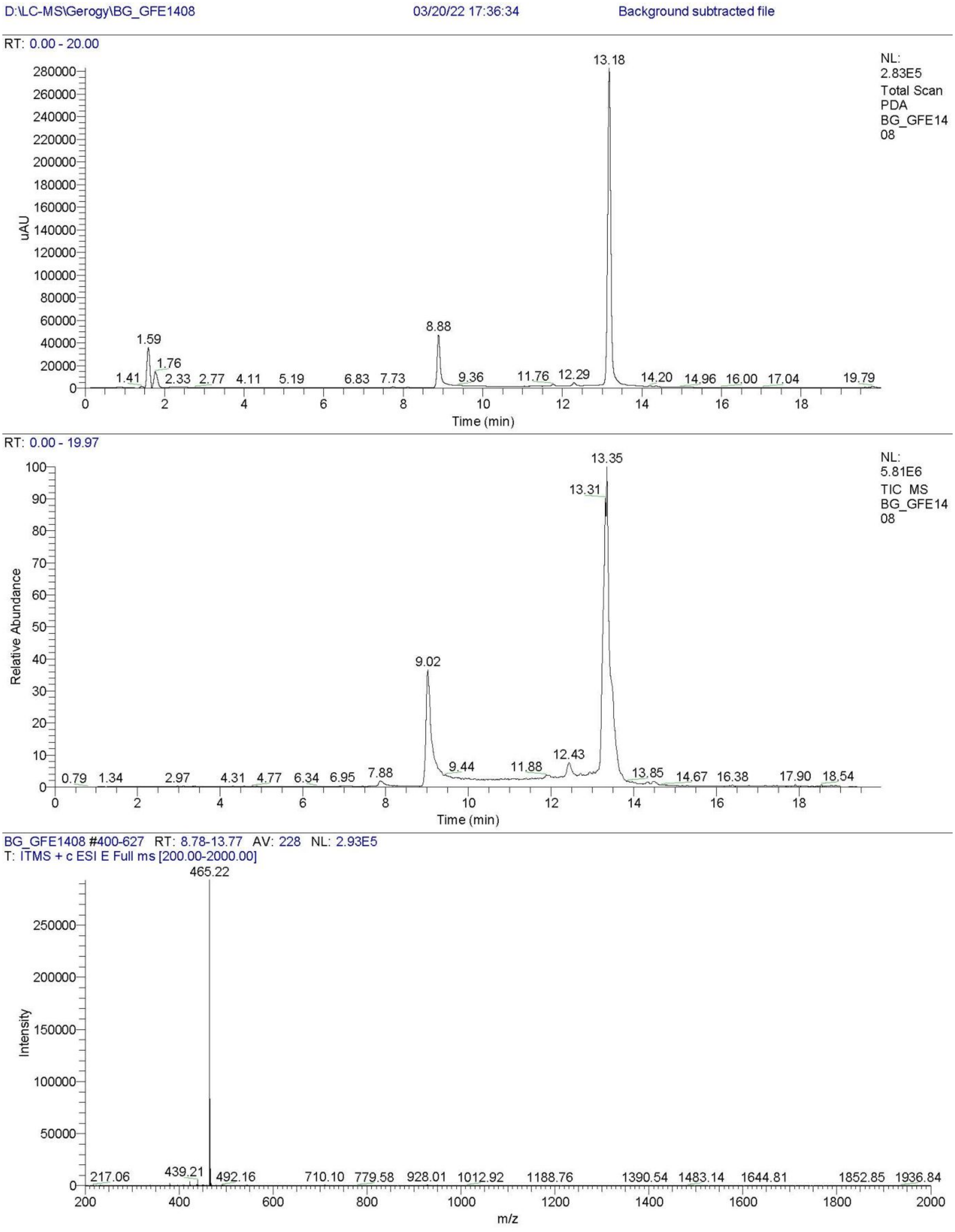

Figure S30. LC-MS data for SP-6.

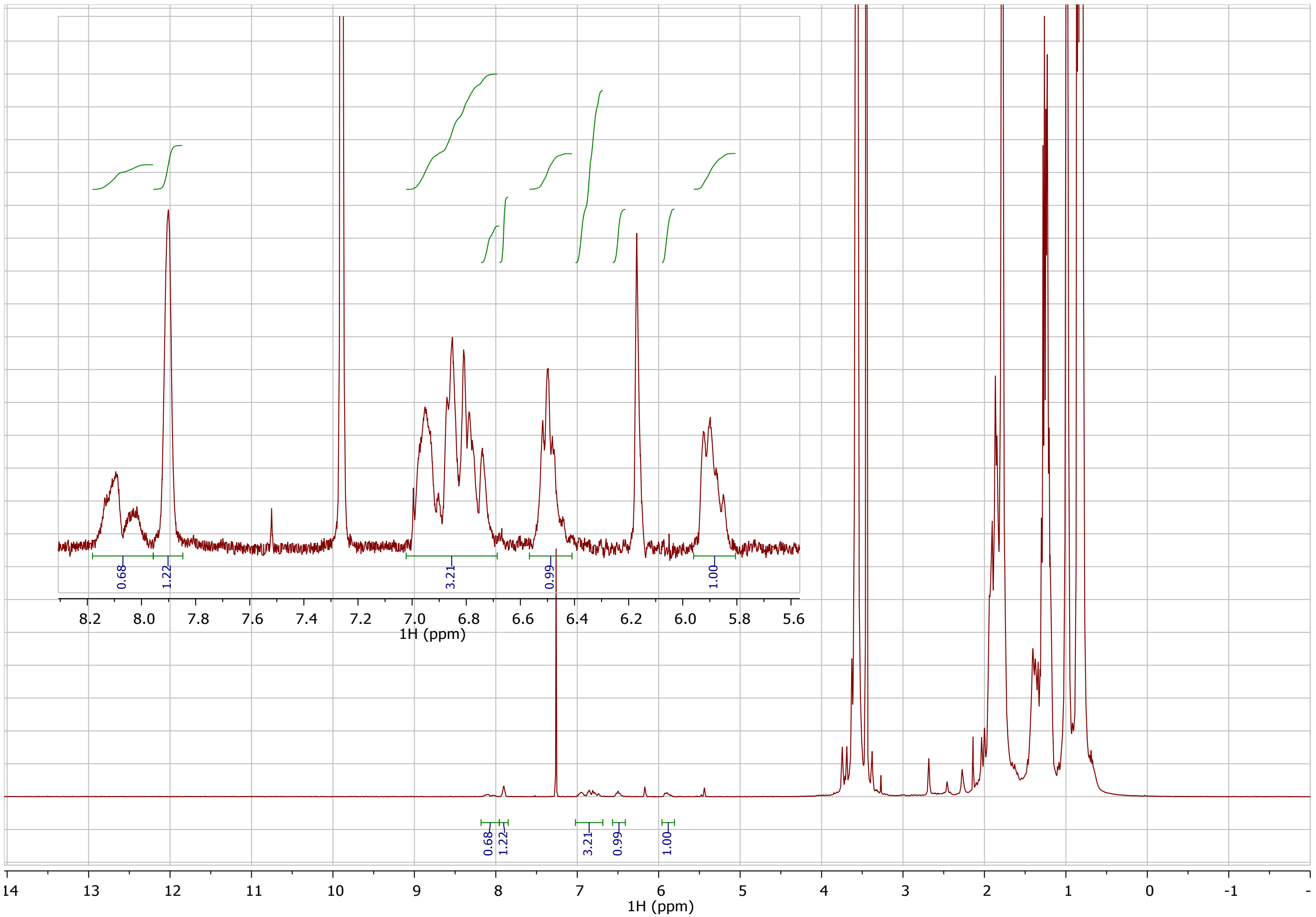

Figure S31. ^{1}H NMR spectrum for p-SC ($CDCl_3$).

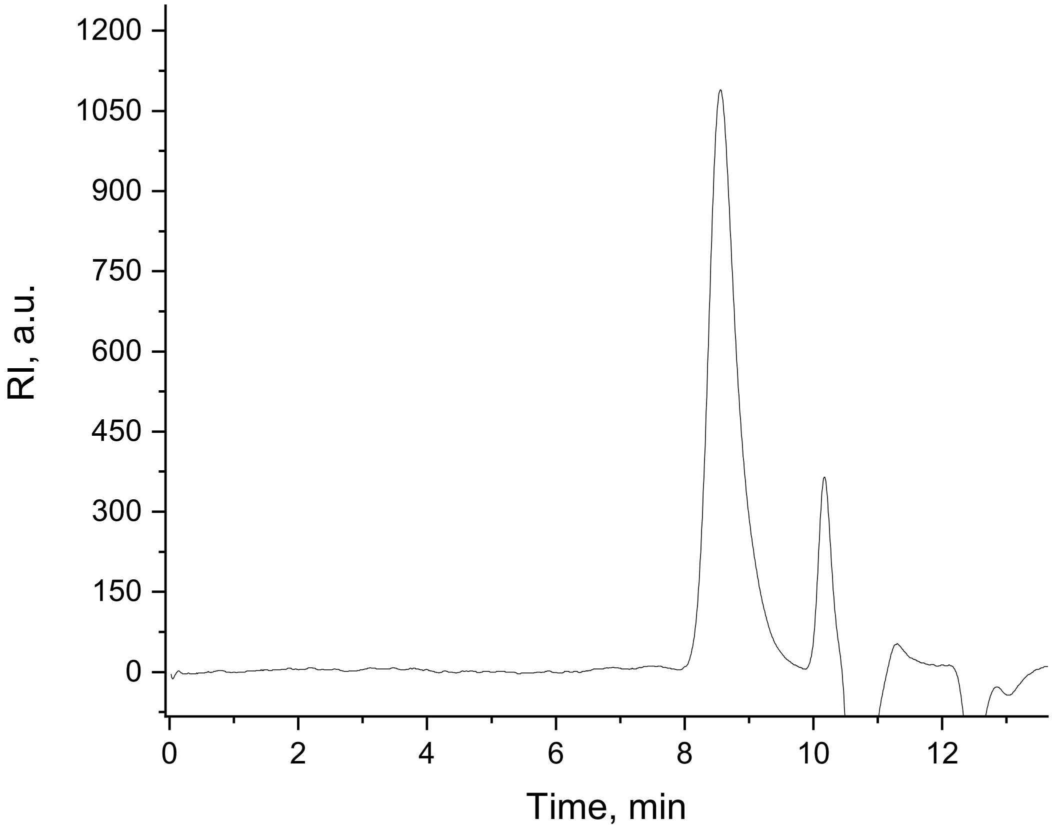

Figure S32. GPC data for p-SC.

3. Spectroscopy and imaging

Spectroscopy: data collection

Cast polymer samples on the glass cover slips were incubated at 125°C for 10 min and spectroscopy measurements were performed immediately after. The samples were (1) thermally equilibrated until no change in the transmittance spectrum occurred, (2) irradiated using 375 nm (all samples but p-SC) or 405 nm (p-SC) LED (Thorlabs, full power, 2 cm distance from the sample), and (3) the data acquisition started immediately after the LEDs were switched off; the lag time between data acquisition start and the disappearance of the UV light can be seen in Figure S37 for high temperature measurements with small time delays between spectra. After duplicate measurements were performed, the temperature was lowered and a new set of measurements was collected.

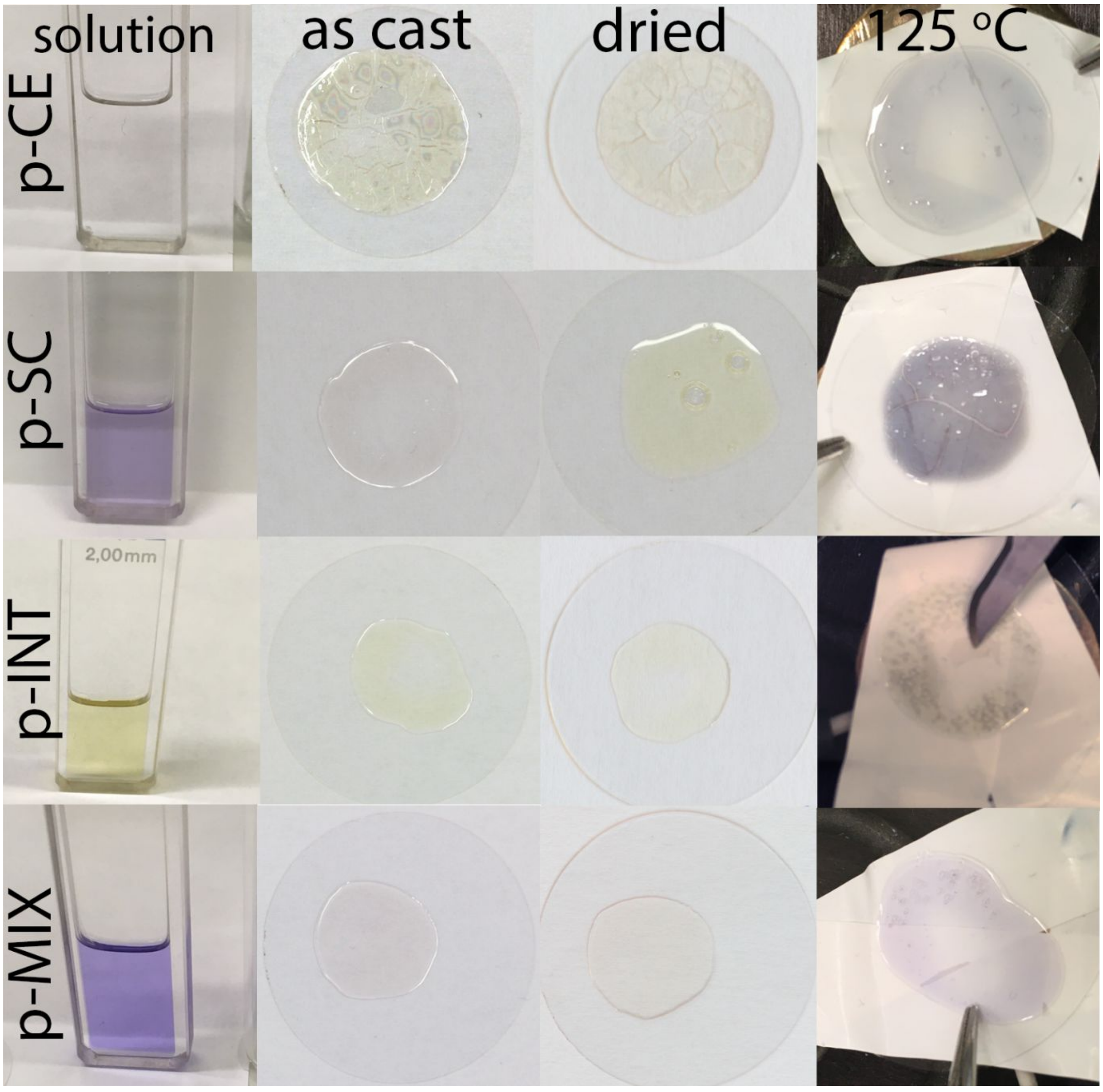

Figure S33. Color progression for samples with covalently attached SP during dissolution, casting, and annealing. Photographs show samples immediately after casting and ambient drying (as prepared), after vacuum drying, and heating at 125°C. Photographs of dried samples under white light are shown in the main Figure 2. Note the coloration of p-SC and p-MIX are different from the p-CE and p-INT.

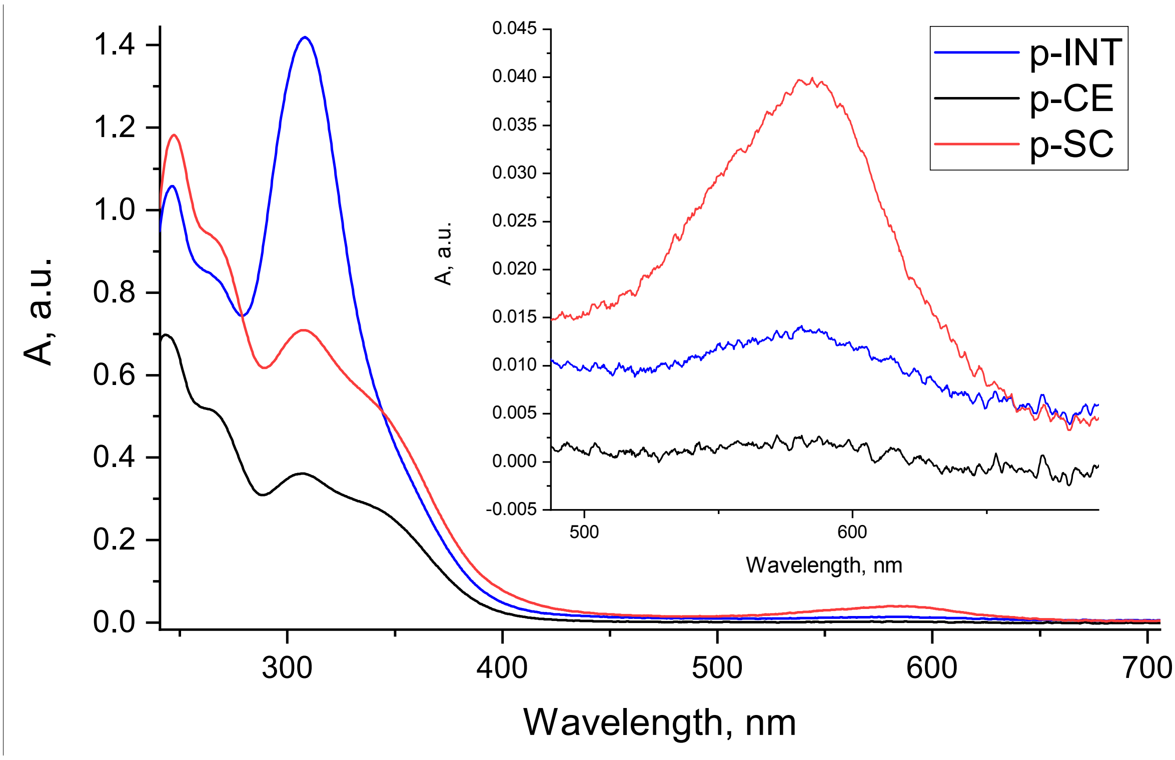

Figure S34. UV-VIS absorption spectra of samples with covalently attached SP in $CHCl_3$ solution. Concentrations: 7.9 mg/ml (p-INT), 6 mg/ml (p-CE), 5.3 mg/ml (p-SC), 2 mm optical path.

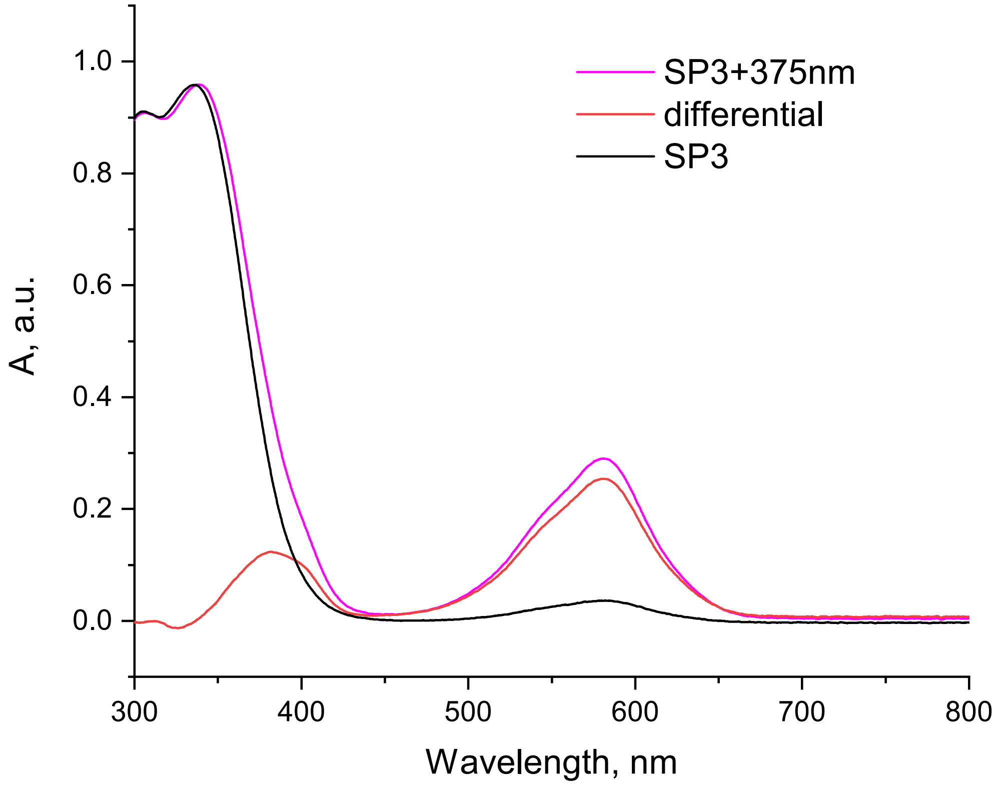

Figure S35. UV-VIS absorption spectra of **SP-3** in $CHCl_3$ at room temperature before (black) and after (magenta) irradiation with UV light (375 nm). Note the significant change of the spectrum upon MC formation (differential spectra in red). Concentrations: 0.626 mM, 2 mm optical path.

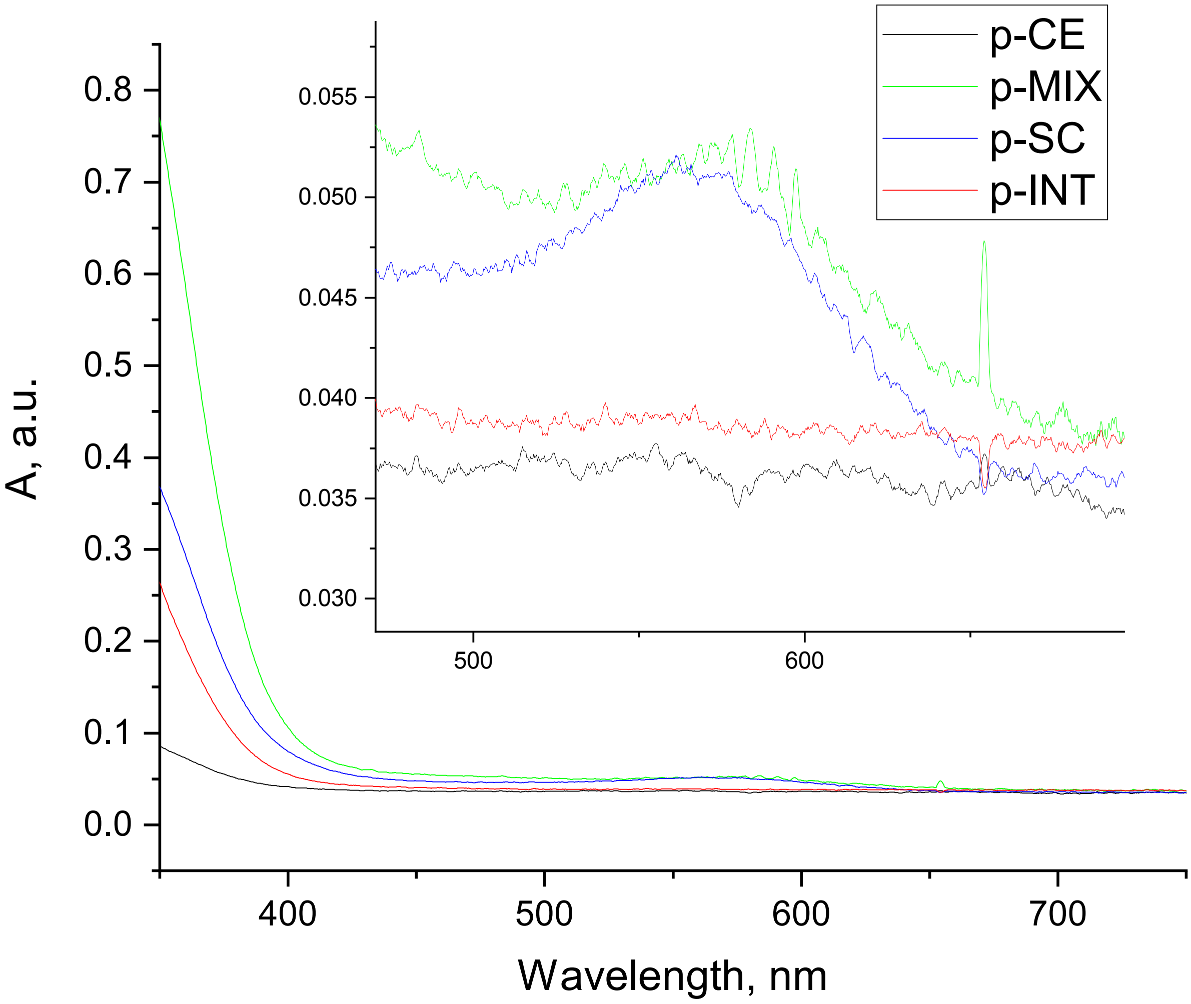

Figure S36. UV-VIS absorption spectra of as cast film samples. See photographs in Figure S33 above for reference.

Spectroscopy: data processing

The transmittance evolution curves collected as described above at different temperatures were converted to absorbance units. A fixed wavelength (573 nm) was selected for plotting the absorbance as a function of time and fitted to a mono-exponential decay function. The rate constants were extracted from the fits and used to estimate the activation barrier using the Arrhenius equation. All plots are shown in the main Figure 3. Figures S37 and S38 below depict an example of the step-wise processing routine for the **p-MIX** sample.

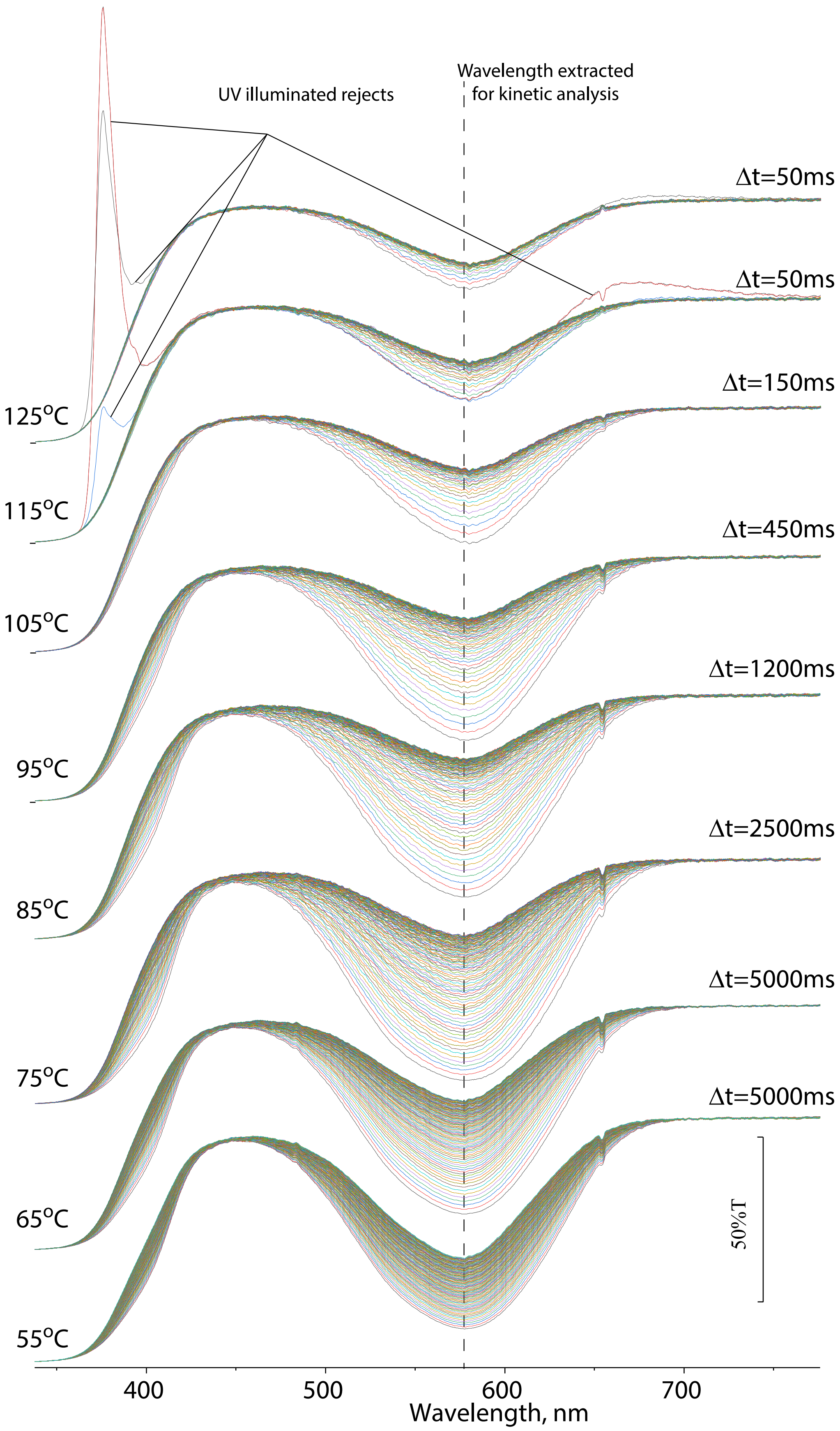

Figure S37. Stacked transmittance spectra for p-MIX. Temperatures and time delays between spectra are indicated on the graphs. Transmittance values were converted to absorbance units for further analysis.

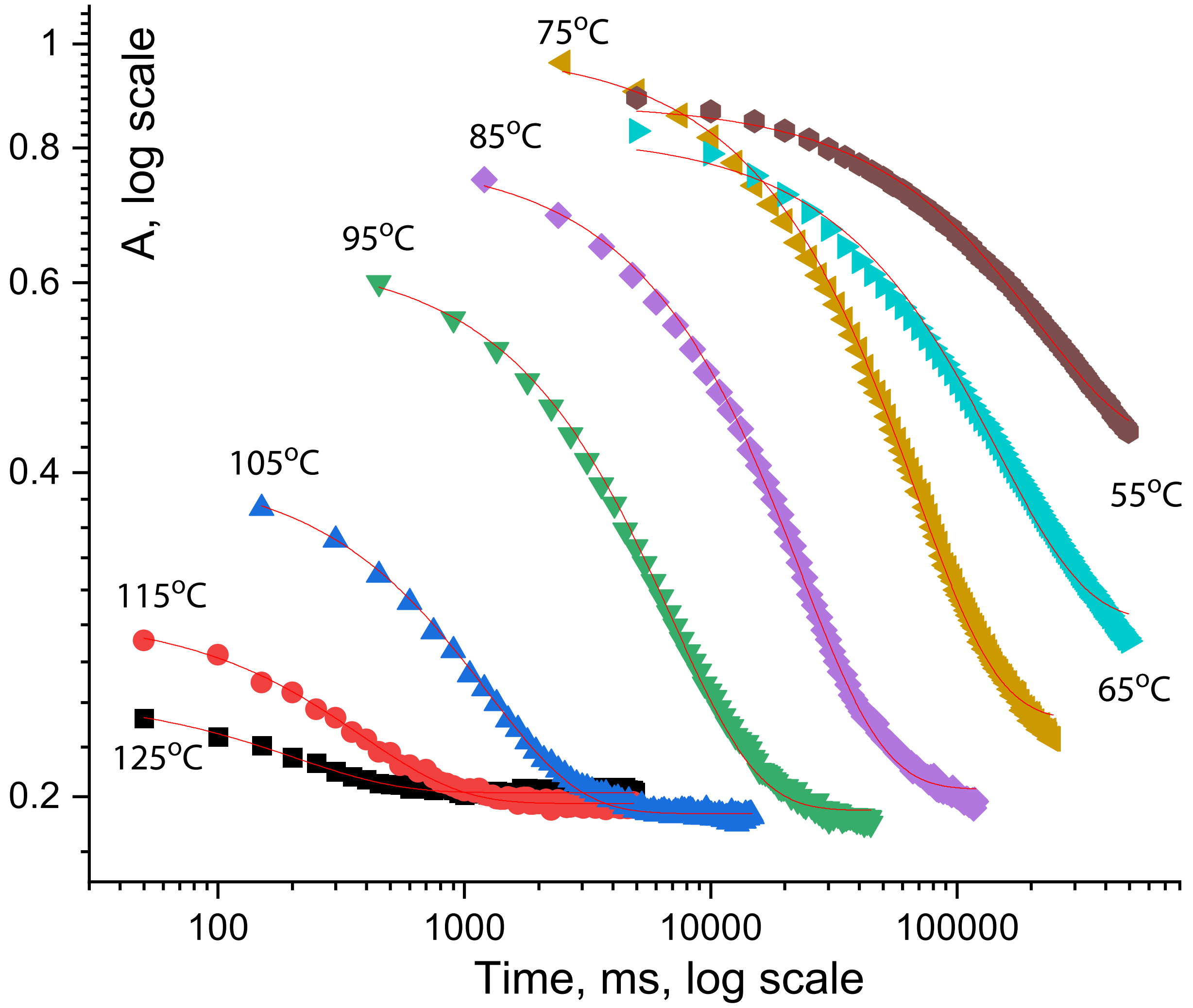

Figure S38. Absorbance evolution at different temperatures for the p-MIX sample. Red lines depict mono-exponential fits of $A = A_0 + B*\exp(-t/\tau)$ to the data, where τ corresponds to the re-equilibration lifetime and the rate constant is derived as $k = \tau^{-1}$.

Table S1. Rate constants used for Arrhenius equation fitting; ln (*k*) values were extracted from kinetic curve fitting. Data correspond to two replicates for p-MIX, p-INT, and p-SC, and to three replicates for p-CE. *The last 50% of the conversion curve was used for p-SC.

	**p-CE**			**p-MIX**		**p-INT**		**p-SC***	
**Replicate**	1	2	3	1	2	1	2	1	2
**Temp [°C]**	ln(k)	ln(k)	ln(k)	ln(k)	ln(k)	ln(k)	ln(k)	ln(k)	ln(k)
125	-4.75918	-4.92733	-5.03305	-5.25227	-4.08598	-5.98896	-5.48272	-5.78383	-6.65673
115	-5.772	-5.94319	-6.01141	-5.80694	-5.18739	-6.72841	-6.3761	-7.08087	-7.91717
105	-6.80814	-7.0934	-7.31588	-6.86066	-6.62672	-7.67925	-7.33934	-8.29004	-9.31326
95	-7.99396	-8.24722	-8.42989	-8.44204	-7.90765	-8.52238	-8.41923	-9.93687	-11.3155
85	-9.20382	-9.48098	-9.84634	-9.59492	-9.1327	-9.42384	-9.30064	-11.7408	-13.1412
75	-10.3994	-11.0259	-11.1119	-10.642	-10.2326	-10.2557	-9.83473	-13.9858	-14.7638
65	-11.5698	-11.5984	-11.8481	-11.5364	-10.8373	-10.6191	-10.3169	-	-
55	-12.6835	-12.5188	-12.5503	-12.024	-12.1808	-11.0068	-10.8558	-	-
45	-	-	-	-	-	-11.4458	-11.4885	-	-

NOTE: We noted no significant deviation when measuring each individual sample in several cycles; therefore, two or three sample replicates were used to acquire the representative averages.

Statistical analysis and E_{act} calculation

To statistically validate the observed changes in the activation barrier (see also main Figure 3B), the rate constants (Table S1) that resulted from the Arrhenius equation fits of the different samples were subjected to an F-test.[4] The null hypothesis with only one linear regression (H0) was tested against a model with two linear regressions (H1). In all cases, the null hypothesis was rejected with significant probability ($p<0.05$). The activation energies E_{act} were calculated from the slopes of the Arrhenius plots.

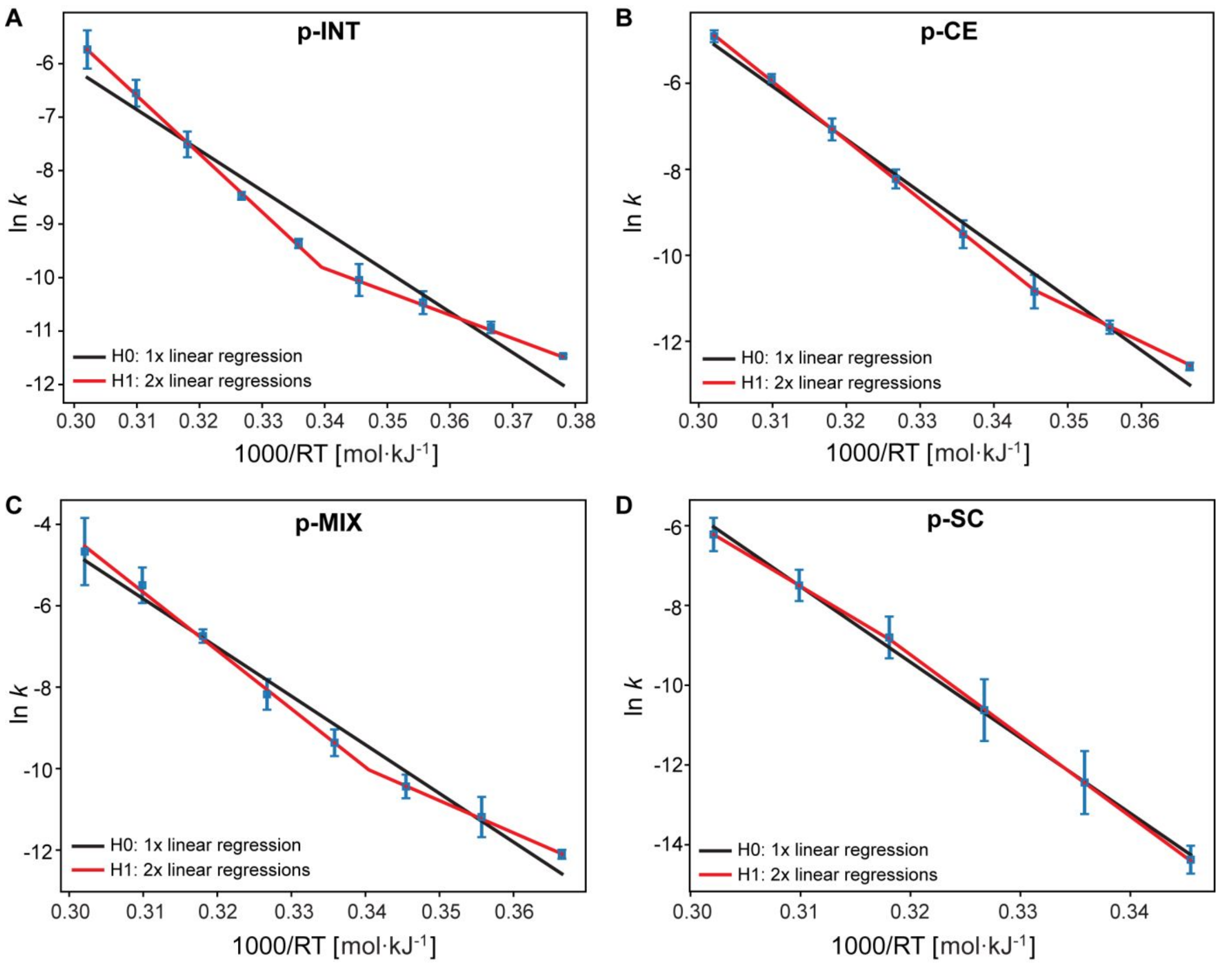

Figure S39. Arrhenius plots of rate constants with applied fits of one linear regression (H0) and two, piecewise linear regressions (H1) for statistical F-test analysis using 95% confidence level for null hypothesis (H0) rejection. Statistical test values of samples (**A**) p-INT: F-value = 428.19, P-value = 2.566e-6; (**B**) p-CE: F-value = 332.151, P-value = 3.58e-5; (**C**) p-MIX: F-value = 25.07, P-value = 0.00545; and (D) p-SC: F-value = 39.95, P-value = 0.0244. E_{act} values for all samples are denoted in the main Figure 3B.

Imaging: setup and data collection

The cast polymer samples on glass cover slips were prepared prior imaging in the same way as performed for spectroscopy (vacuum drying followed by annealing at 125°C). After annealing, the samples were equilibrated to 65°C – a temperature at which the merocyanin (MC) form concentration decays with sufficient rate to be captured within several minutes. All measurements were performed at 65°C.

Fluorescent imaging was performed using a Nikon Eclipse E600 microscope, equipped with a 10x objective (Nikon LU PLAN 10x/0.30 WD EPI). Sample excitation was performed via a xenon lamp in combination with a bandpass filter (Thorlabs, 532/15) to generate green light for excitation. The light emission of the sample was passed through a long-pass filter (Thorlabs, 590LP) and collected with a CCD camera (Nikon DS-Ri1). The gain and exposure were adjusted to produce ca. 5-10 k counts per pixel (maximal range of 65k counts for the used CCD). Before every measurement sample was irradiated with a 375 nm LED to perturb the SP-MC equilibrium and time lapse was recorded immediately after to register the thermal conversion of the MC fluorophore. Caution was made to ensure that every part of the image remained within the dynamic range of the CCD detector below 65 k counts.

Imaging: analysis

The time lapse image stacks were analyzed *via* a self-written script in Igor Pro (V6.37, Wavemetrics, USA). The background correction was performed using the last image of the image stacks, which corresponds to a thermally relaxed sample near equilibrium. The emission intensity over time for each pixel was extracted from the time lapse image stacks and fitted to a mono-exponential decay function to determine the decay lifetime parameter τ per pixel, which were then used to construct a decay lifetime heat map of the sample.

References

[1] T. A. Kim, M. J. Robb, J. S. Moore, S. R. White, N. R. Sottos, *Macromolecules* **2018**, *51*, 9177-9183.

[2] J. T. Lai, D. Filla, R. Shea, *Macromolecules* **2002**, *35*, 6754-6756.

[3] J. S. Moore, S. I. Stupp, *Macromolecules* **1990**, *23*, 65-70.

[4] O. Sureiman, C. Mangera, *Journal of the Practice of Cardiovascular Sciences* **2020**, *6*, 116-122.
